# Anthocyanin gene enrichment in the distal region of cotton chromosome A07: mechanisms of reproductive organ coloration

**DOI:** 10.3389/fpls.2024.1381071

**Published:** 2024-04-18

**Authors:** Liuchang Zheng, Jilong Zhang, Haiyan He, Zhigang Meng, Yuan Wang, Sandui Guo, Chengzhen Liang

**Affiliations:** ^1^ College of Life Sciences, Qingdao Agricultural University, Qingdao, China; ^2^ Biotechnology Research Institute, Chinese Academy of Agricultural Sciences, Beijing, China

**Keywords:** cotton, anthocyanin, MYB, GST, metabolic gene clusters

## Abstract

**Introduction:**

The biosynthesis of secondary metabolites like anthocyanins is often governed by metabolic gene clusters (MGCs) in the plant ancestral genome. However, the existence of gene clusters specifically regulating anthocyanin accumulation in certain organs is not well understood.

**Methods and results:**

In this study, we identify MGCs linked to the coloration of cotton reproductive organs, such as petals, spots, and fibers. Through genetic analysis and map-based cloning, we pinpointed key genes on chromosome A07, such as *PCC/GhTT19*, which is involved in anthocyanin transport, and *GbBM* and *GhTT2-3A*, which are associated with the regulation of anthocyanin and proanthocyanidin biosynthesis. Our results demonstrate the coordinated control of anthocyanin and proanthocyanidin pathways, highlighting the evolutionary significance of MGCs in plant adaptation. The conservation of these clusters in cotton chromosome A07 across species underscores their importance in reproductive development and color variation. Our study sheds light on the complex biosynthesis and transport mechanisms for plant pigments, emphasizing the role of transcription factors and transport proteins in pigment accumulation.

**Discussion:**

This research offers insights into the genetic basis of color variation in cotton reproductive organs and the potential of MGCs to enhance our comprehension of plant secondary metabolism.

## Introduction

In plants, the genomic architecture for the biosynthesis of secondary metabolites is characterized by the organization of relevant genes into metabolic gene clusters (MGCs). These clusters facilitate the synthesis of critical compounds that enhance plant resilience against diverse biotic stresses (Jonczyk et al., 2008; Chu et al., 2011; Sue et al., 2011; Takos et al., 2011; Guo et al., 2017).

Plant MGCs can be classified into two distinct configurations. The first configuration dominantly is tightly packed gene clusters, wherein genes responsible for both the initial and subsequent enzymatic reactions are sequentially arranged. This arrangement is exemplified by the thalianol gene cluster in *Arabidopsis* and the diterpenoid gene cluster in rice, illustrating a streamlined genomic strategy for secondary metabolite biosynthesis (Liu et al., 2020; Zhan et al., 2020). The second configuration involves partially adjacent and dispersed gene clusters, where a core group of genes is closely located, albeit with several genes situated distantly. The steroidal alkaloid biosynthesis in tomatoes follows the α-tomatine pathway, controlled by a gene cluster on chromosome 7. This cluster includes two 2-oxoglutaratedependent dioxygenase (*GAME11* and *GAME6*)genes, four genes encoding glycosyltransferase (*GAME1*,*GAME17*,*GAME18* and *GAME2*), and the cytochrome P450 monooxygenase gene (*GAME7*), separated by 8 Mb from the nearest gene cluster (Itkin et al., 2013). Similarly, cucumber’s cucurbitacin biosynthesis is managed by a gene cluster on chromosome 6, with additional cytochrome P450 (CYP) genes on chromosomes 3 and 1, indicating a spread but functionally unified genomic organization (Shang et al., 2014). In potatoes, sterol biosynthesis genes are found on chromosomes 7 and 12, highlighting the varied arrangement of MGCs in different plant species (Cardenas et al., 2016).

The biosynthesis pathways of anthocyanins and proanthocyanidins may also be regulated by the presence of MGCs, underscore the evolutionary significance of such genomic configurations for plant adaptation and survival. Anthocyanins, alongside carotenoids and betalains, are pivotal for the vibrant pigmentation observed in plant tissues, with anthocyanins occupying a major role in coloration (Tanaka et al., 2008). These pigments are predominantly accumulated in essential reproductive structures—flowers, fruits, and seeds—creating diverse, vivid color patterns that both attract pollinators and confer resistance to biotic and abiotic stressors (Stintzing and Carle, 2004; Duan et al., 2014). Anthocyanin biosynthesis is mediated by a sequence of enzymatic reactions on the endoplasmic reticulum (ER), initiating from phenylalanine (Winkel, 2004; Yang et al., 2018; Belwal et al., 2020). Meanwhile, this pathway further promoted the synthesis of proanthocyanidins (PAs), which affected the pigmentation across various plant organs (Shi et al., 2021; Zhao et al., 2023).

Genes involved in the biosynthesis of anthocyanins and proanthocyanidins have been identified across various plant species (Winkel-Shirley, 2001; Ma et al., 2021; Luan et al., 2022; Yang et al., 2022; He et al., 2023). The anthocyanin biosynthetic pathway encompasses three main steps: Initially, phenylalanine is converted into 4-Coumaroyl-CoA via the phenylalanine ammonia-lyase (PAL), cinnamic acid hydroxylase (C4H), and 4-coumarate-CoA ligase (4CL) enzymes. Subsequently, 4-Coumaroyl-CoA is transformed into dihydroflavonol, a shared precursor for both anthocyanins and proanthocyanidins, through the action of chalcone synthase (CHS), chalcone isomerase (CHI), and flavanone 3-hydroxylase (F3H). Dihydroflavonol is then converted into the colorless leucoanthocyanidins (leucopelargonidin, leucocyanidin, and leucodelphinidin) by dihydroflavonol reductase (DFR). In the final synthesis phase, anthocyanins are produced from these colorless precursors via anthocyanin synthase (ANS) and leucoanthocyanidin dioxygenase (LODX), with subsequent glycosylation and acylation by UDP-3-O-glucosyltransferase (UFGT) to yield stable anthocyanins. Alternatively, colored anthocyanins can be reduced to epicatechins by anthocyanin reductase (ANR), or directly to catechins by leucoanthocyanidin reductase (LAR), which then polymerize to form proanthocyanidins in the vacuole (Zhao et al., 2010; Yu et al., 2023). The activity of these enzymes is regulated by v-myb avian myeloblastosis viral oncogene homolog (MYB), WD40, and basic Helix-Loop-Helix (bHLH) transcription factors, highlighting a complex control mechanism over the biosynthetic pathway (Petroni and Tonelli, 2011; Yan et al., 2021; Shi et al., 2023).

The transport of anthocyanins, catechins, and proanthocyanidins to vacuoles can occur through two pathways: vesicle transport facilitated by vesicle wrapping and transport proteins, including ATP-binding cassette transporters (ABC/MRP), multidrug and toxic compound extrusion (MATE), multidrug resistance-associated protein (MRP), and glutathione S-transferase (GST) (Grotewold and Davies, 2008; Gomez et al., 2011). GST proteins play a crucial role in the transport of anthocyanins and monomers of anthocyanins. Acting as transport carriers, GST proteins directly bind to anthocyanins or precursors of proanthocyanidins in the cytoplasm, facilitating their transport to the vacuolar membrane. Subsequently, these compounds are translocated across the membrane into the vacuole after recognition by ABC/MRP or MATE transmembrane proteins located on the vacuolar membrane (Loyall et al., 2000; Zhao, 2015).

The genus *Gossypium*, encompassing over fifty species, exhibits significant diversity in flower and fiber coloration, primarily attributed to differential gene expression within the anthocyanin/proanthocyanidin biosynthetic pathways (Tan et al., 2013). Notably, key genes implicated in the regulation of these pathways, such as *G. barbadense Beauty Mark* (*GbBM*), *G. hirsutum TRANSPARENT TESTA 2*–*3A* (*GhTT2-3A*) in reproductive tissues, are members of the MYB transcription factor family (Yan et al., 2018; Abid et al., 2022). Additionally, the glutathione S-transferase gene *GhTT19* plays a pivotal role in the transport of anthocyanins/proanthocyanidins, influencing flower petal pigmentation (Chai et al., 2023). Intriguingly, these genes associated with the synthesis and transports of anthocyanins/proanthocyanidins in cotton reproductive organs are clustered in the distal region of chromosome A07 in cotton. The enrichment of these genes in specific chromosomal regions related to anthocyanin synthesis and transport remains largely unexplored. Our study demonstrates a significant correlation between the distal region of chromosome A07 and the pigmentation of cotton reproductive organs, suggesting the formation of a metabolic gene cluster that regulates the anthocyanin pathway, thereby influencing coloration. This chromosomal configuration remains conserved across the transition from diploid to tetraploid species, underscoring its importance in cotton reproductive development and propagation. These insights offer a foundation for future research aimed at enhancing cotton hybrid breeding, the cultivation of naturally colored fibers, and improving cotton yield.

## Materials and methods

### Plant materials

The *G. barbadense* line HaiR, *G. hirsutum* line Y18R, and red color cotton line *pcc*, were used in this study. The red-petaled *pcc* cotton was selected from over 1,000 varieties in the cotton germplasm resource library. The Y18R line is characterized by white flowers, whereas the *pcc* line exhibits red flowers. The HaiR line is distinguished by its yellow flowers. For the generation of F_2_ mapping populations, HaiR and *pcc* were selected as the parental lines.

### Cotton planting conditions

Cotton plants were grown under field conditions at the High-Tech Industry Park of the Chinese Academy of Agricultural Sciences in Langfang, Hebei Province (N39°520, E116°700E). Photographic documentation and petal collection were conducted during and post-flowering for subsequent analyses. Quantitative Reverse Transcription Polymerase Chain Reaction (qRT-PCR) and anthocyanin content determination were performed, with each assay conducted in triplicate.

### Map-based cloning

Map-based cloning was conducted using an F_2_ population consisting of a total of 870 cotton plants, which exhibited two distinct phenotypes: yellow-flowered and red-flowered. For genetic linkage analysis, 270 individuals displaying the yellow color flower phenotype were selected at the flowering stage. A total of 720 pairs of insertion/deletion (InDel) primers, designed based on genome-wide InDel markers differentiating *G.barbadense* and *G.hirsutum* genotypes, were used for preliminary gene mapping (He et al., 2018). The PCR amplifications were electrophoresed on a 4% agarose gel. Primers utilized for fine mapping are detailed in **Supplementary Table 3**.

### Draw the anthocyanin metabolism pathway

Building on previous research into the metabolic pathways of anthocyanins and proanthocyanidins (Liu et al., 2021; Pucker and Selmar, 2022; Sunil and Shetty, 2022), and we made minor adjustments and illustrated these pathways using Figdraw to accurately depict the metabolic regulation model.

### RNA extraction and qRT-PCR analysis

For expression analysis, samples were collected and immediately flash-frozen in liquid nitrogen, then stored at -80°C until RNA extraction. Total RNA was extracted using the RNAprep Pure Plant Kit (TIANGEN, DP441, Beijing, China) following the manufacturer’s protocol. cDNA synthesis was performed using the TransScript One-Step gDNA Removal and cDNA Synthesis SuperMix (TRAN, AT311, Beijing, China) as per the manufacturer’s guidelines. qRT-PCR analysis was conducted using the Chromo 4 Real-Time PCR Detection System (Bio-Rad, CFX96, Hercules, USA), adhering to the manufacturer’s instructions. The cotton *GhHistone* gene served as the internal reference. Experiments were independently replicated three times. Primers for qRT-PCR are detailed in **Supplementary Table 3**. The relative expression levels of genes were quantified using the 2^-ΔΔCT^ method (Livak and Schmittgen, 2001).

### Gene expression analysis

The raw RNA-seq datasets for red and white flowers of *G. hirsutum* were obtained from PRJNA878950, while those for brown and white cotton fibers were sourced from PRJNA766762. Following the removal of low-quality reads, the remaining clean reads were aligned to the TM-1 reference genome using Hisat2 software (Kim et al., 2019). Differential gene expression analysis was conducted using featureCounts for the calculation of Transcripts Per Million (TPM) values (Liao et al., 2014; Zhao et al., 2020). Gene expression heatmaps were generated utilizing the Pheatmap package (Hu, 2021).

### Anthocyanin analysis

The determination of anthocyanin content followed the methodology described by Xu et al. (2012). Petal samples (0.1 g) were retrieved from a -80°C freezer, ground to a powder in liquid nitrogen, and then mixed with 1 mL of methanol. The mixture was vortexed thoroughly and sonicated for 15 minutes. After centrifugation at 12,000 rpm for 10 minutes, the supernatant was diluted tenfold with methanol and filtered through a 0.22 μm organic membrane. LC-MS analysis was performed using a Thermo U3000 HPLC System. The peak area of the mixture was quantified by comparison with the peak areas of standard substances, including cyanidin, delphinidin, and pelargonidin (all from Sigma, USA). The formula for calculating anthocyanin content is as follows: [A = (C2 - C1) * V * N where (A) represents the content of various anthocyanins in the sample (mg/kg), (C2) is the concentration of each anthocyanin in the test solution (mg/L), (C1) is the concentration of each anthocyanin in the blank solution (mg/L), (V) is the volume of the solution (mL), and (N) is the dilution factor.

### Population structure analysis

Total 96 accessions derived from four projects (PRJNA349094, PRJNA680449, PRJNA412456, PRJNA720818) were analyzed (see **Supplementary Table 4**). The sequencing reads were aligned to the *G. hirsutum* TM-1 reference genome using BWA software (Li and Durbin, 2009). The resulting BAM files were processed for sorting and filtering using SAMtools (version 1.15) with default parameters (Danecek et al., 2021). SNP calling at the population level was conducted using GATK software (version 4.0) (Van der Auwera et al., 2013), generating a GVCF file for each sample. These GVCF files were then merged, applying the following parameters for filtering: –filter-expression “QD < 2.0 || MQ < 40.0 || FS > 60.0 || SOR > 3.0 || MQRankSum < -12.5 || ReadPosRankSum < -8.0” –filter-name ‘SNP_filter’.

A total of 120,126 SNPs, with a minor allele frequency (MAF) > 0.01 and missing data < 10%, were identified using Plink (version 1.9). These SNPs were used to construct a phylogenetic tree using the neighbor-joining method in PHYLIP software (version 3.698) (Retief, 2000). The phylogenetic tree was visualized and refined using iToL (https://itol.embl.de/). Population genetic structure was assessed using Admixture (Version 1.3.0), with the number of genetic clusters (K) ranging from 2 to 6 and each run consisting of 10,000 iterations.

### Analysis of the domestication selection

Using VCFtools (version 0.1.16) (Danecek et al., 2011), we calculated fixation index (*Fst*) and nucleotide diversity (π) employing a sliding window approach. Each window spanned 50kb, with successive windows overlapping by 10kb. This methodology facilitates a detailed and nuanced analysis of genetic differentiation and diversity across the genome.

### Identification of MYB and GST gene families

Genomic sequences, annotation files, and protein sequences for *Gossypium* species including *G. herbaceum* (A1), *G. arboretum* (A2), *G. raimondii* (D5), *G. hirsutum* (AD1), *G. barbadense* (AD2), *G. tomentosum* (AD3), *G. mustelinum* (AD4), and *G. darwinii* (AD5) were retrieved from Phytozome (Goodstein et al., 2012) and CottonMD (Yang et al., 2023). Protein sequences for the GST and MYB gene families were downloaded from the TAIR database (https://www.arabidopsis.org/). Hidden Markov Model (HMM) profiles for GST_C (PF00043), GST_N (PF02798), and MYB (PF00249) were acquired from the Pfam database (version 35.0).

To identify homologs of the MYB and GST gene families across the *Gossypium* species, *Arabidopsis thaliana* GST and MYB protein sequences served as query sequences in BLASTp searches (version 2.15), with stringent criteria set at e-values < 1e-10 and identity ≥ 30%. Concurrently, HMMER (version 3.4) software, utilizing the hmmersearch function and the aforementioned HMM profiles, was employed to search for protein sequences within the cotton varieties, applying an e-value threshold of ≤ 1e-5. Results from both BLASTp and hmmersearch were integrated, allowing for the exclusion of structurally incomplete sequences from the GST and MYB gene families in the analyzed *Gossypium* species.

### Multiple sequence alignment and phylogenetic tree construction

Protein sequences from various *Gossypium* species were consolidated and subjected to multiple sequence alignment using MUSCLE (version 5.0). Subsequently, a multi-species evolutionary tree was constructed employing the Maximum Likelihood (ML) method via FastTree (Price et al., 2009). The resulting evolutionary tree was visualized and refined using the online tool iTOL (Letunic and Bork, 2021). Within this phylogenetic framework, we specifically identified protein sequences associated with anthocyanin biosynthesis. These sequences were then realigned using MUSCLE (version 5.0), and the alignment was further refined for accuracy and clarity using GeneDoc.

### Chromosomal localization, duplication, and synteny analysis

Protein sequences from all varieties were compiled into a database, with each serving as its own query sequence. For sequence alignment, Diamond (version 2.1.8) (Buchfink et al., 2021) was utilized, applying an e-value threshold of < 1e-10. The gene annotation files were then converted into GFF format. Duplication classification of genes was conducted using McscanX (Wang et al., 2012), which produced source files detailing gene family duplications. A Perl script, get_mapchart_tandem.pl, was used to generate configuration files for MapChart (Voorrips, 2002), which depicted the genomic locations and tandem duplications of the GST and MYB gene families on chromosomes.

For inter-species collinearity analysis, MCScanX was used to create collinearity files. These files were then modified using another Perl script, highlight.pl, to emphasize blocks containing GST and MYB genes. The visualization of these results was achieved through the JCVI software (Tang et al., 2008), effectively mapping the positions and tandem duplications of the GST and MYB gene families, as well as highlighting significant collinearity blocks.

### Construct protein regulatory network

To elucidate the relationship between MYB genes on Chromosome A07 and structural genes involved in anthocyanin and proanthocyanidin biosynthesis, we employed the String database (https://string-db.org/) for protein-protein interaction (PPI) prediction. The resultant PPI predictions were then visualized through the construction of a network diagram using Cytoscape software (version 3.10.1) (Otasek et al., 2019).

### Data analysis

To compare two groups, the Student’s t-test was utilized. For analyzing significance across multiple groups, one-way ANOVA followed by Tukey’s multiple comparisons test was employed. Data are expressed as mean ± standard deviation (SD), based on three independent biological replicates. Significant differences were denoted as follows: *P < 0.05, **P < 0.01, and ***P < 0.001. All graphs and statistical analyses were performed using GraphPad Prism (version 10.1.2).

## Results

### The distal region of chromosome A07 in cotton regulates genes for reproductive organ coloration

**Figure 1:**
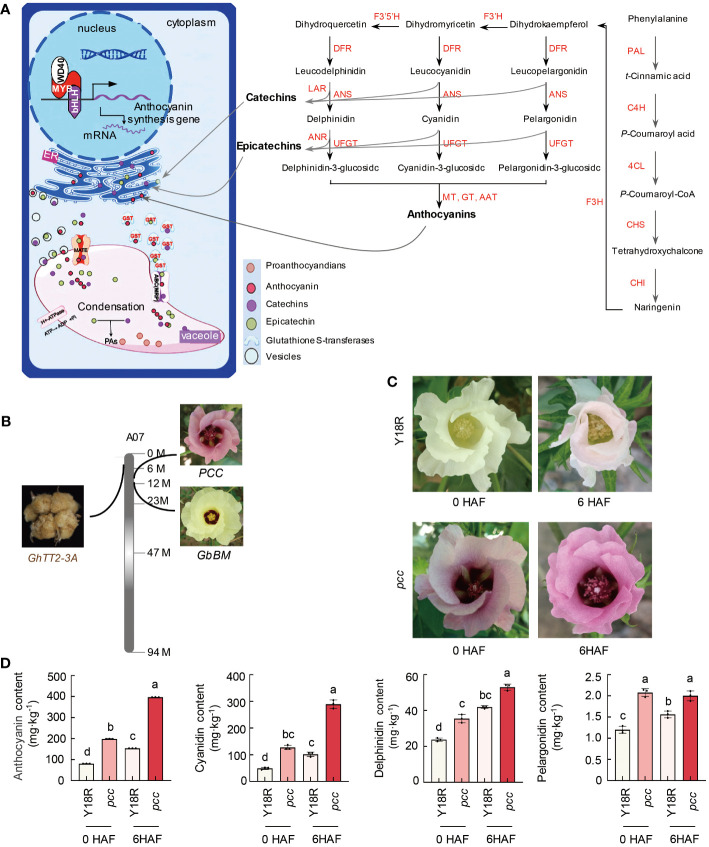
The identification of color genes and a model for anthocyanin biosynthesis and transport. **(A)** The model illustrates the synthesis and transport pathway of anthocyanins and procyanidins in plants. PAL, phenylalanine ammonia-lyase. C4H, cinnamate-4-hydroxylase. 4CL, 4-coumarate-CoA ligase. CHS, chalcone synthase. CHI, chalcone-flavonone isomerase. F3’5’H, flavonoid 3’-monooxygenase. F3’H, flavanone 3ß-hydroxylase. ANR, anthocyanidin reductase. LAR, leucoanthocyanidin reductase. DFR, dihydroflavonol-4-reductase. ANS, anthocyanidin synthase. UFGT, UDP-glucose flavonoid glycosyltransferase. **(B)** Localization of genes related to the color of cotton’s reproductive organs on the distal region of chromosome A07. *PCC*/*GhTT19* is identified as the gene controlling the red color of *G. hirsutum* flowers. *GbBM* is highlighted as a crucial gene influencing the presence of purple spots on *G. barbadense* flowers. *GhTT2-3A* is recognized as the primary gene responsible for the brown color of cotton fibers. **(C)** The flower color of Y18R and *pcc* during and after the flowering stage. HAF, hours after flowering. **(D)** Anthocyanin content during and after the flowering period. Data are shown as mean ± SD from three replicates. Statistically significant differences (*P*<0.05) are indicated by different letters, based on one-way ANOVA analysis. HAF, hours after flowering.

The synthesis of plant anthocyanins is significantly influenced by transcriptional regulatory factors, primarily MYB, bHLH, and WD40, which together form a regulatory system that controls anthocyanin biosynthesis by binding to structural gene promoters. Once synthesized on the endoplasmic reticulum surface, anthocyanins are transported into the vacuole for storage, facilitated by Glutathione S-transferase (GST) and recognized by multidrug resistance associated protein transporter and ABC protein transporter on the vacuolar membrane (**Figure 1A**). We previously reported a gene, *GbBM*, which encodes an R2R3 MYB113 transcription factor. This factor directly targets the promoters of four flavonoid biosynthesis genes (*GbCHS*, *GbDFR*, *GbANS*, and *GbUFGT*), thereby positively regulating the formation of purple spots on cotton flower petals (Abid et al., 2022). In recent years, leveraging map-based cloning techniques, we identified the gene *PCC* from a red-flowered cotton resource, which governs petal coloration in cotton (**Supplementary Figure 1** and **Supplementary Table 1**). *PCC*, previously known as *GhTT19*, encodes a typical glutathione S-transferase. This enzyme is pivotal in petal pigmentation, as it aids in the transport and accumulation of anthocyanins. Significantly, *GbBM* and *PCC/GhTT19* not only share the function of regulating petal coloration but are also positioned closely at the proximal end of the cotton A07 chromosome, separated by a minimal physical distance of merely 0.85MB (**Supplementary Table 2**). This proximity underscores a potentially significant genetic linkage in the control of floral pigmentation.

To investigate the potential association between the proximal region of the cotton A07 chromosome and pigment development, we examined genes responsible for the coloration of reproductive tissues. Notably, our findings revealed that *GhTT2-3A*, a gene implicated in the production of brown fibers through the accumulation of anthocyanins/proanthocyanidins, is also situated in this specific chromosomal region. *GhTT2-3A*, a homolog of the *Arabidopsis* PA regulator TT2 within the R2R3 MYB TF family, triggers the activation of downstream PA structural genes (Yan et al., 2018). This activation facilitates the synthesis and accumulation of proanthocyanidins (PAs) in cotton fibers, leading to the emergence of brown-colored fibers. The physical separation between *GhTT2-3A* and *GbBM* spans 9.2 MB, whereas the gap between *GhTT2-3A* and *PCC/GhTT19* measures 7.6 MB (**Figure 1B** and **Supplementary Table 2**). The genes *GbBM*, *GhTT2-3A*, and *PCC/GhTT19* are closely located on chromosome A07 in cotton, suggesting that the genes responsible for the coloration of cotton reproductive organs likely constitute a metabolic gene cluster at the distal end of chromosome A07.

Further examining the color-determining components, we found that the anthocyanin levels in *pcc* cotton petals, including pelargonidin, cyanidin, and delphinidin, were significantly higher than in white-petaled cotton Y18R, both during and post-flowering. Notably, the pronounced accumulation of cyanidin in *pcc* cotton emerged as the key factor for its red flower phenotype (**Figures 1C, D**). Similarly, the presence of cyanidin, delphinidin, and petunidin was instrumental in forming basal spots on the petals of *GbBM*-induced upland cotton. Additionally, the brown hue of the fibers in brown cotton was predominantly due to the synthesis and buildup of proanthocyanidins.

### Genetic variation and population structure of the distal region of chromosome A07 in cotton

**Figure 2:**
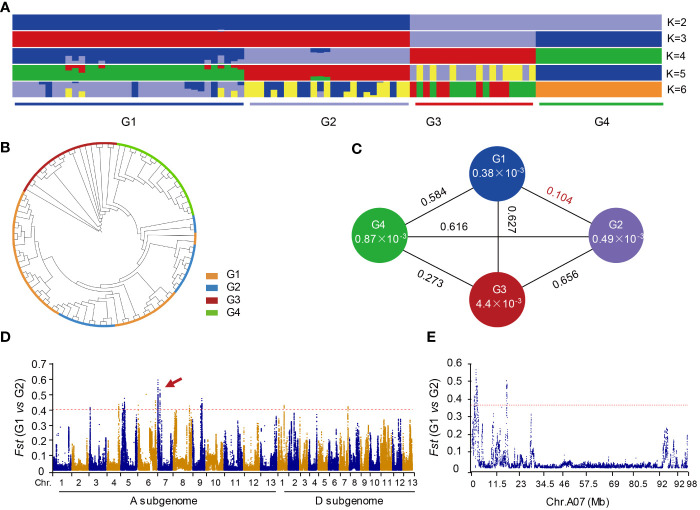
Phylogenetic relationships and genetic differentiation among cotton accessions through a comprehensive analysis. **(A)** The population structure of cotton accessions is depicted, with the accessions categorized into four distinct groups at K = 4. Group G1 comprises *G. hirsutum* accessions characterized by white fiber, while G2 includes *G. hirsutum* accessions with brown fiber. Group G3 is represented by *G. arboreum*, and G4 consists of *G. barbadense* accessions. **(B)** A phylogenetic tree constructed using 120,126 high-quality single nucleotide polymorphisms (SNPs). **(C)** Genetic diversity and population differentiation among the four groups. The nucleotide diversity (π) value for each group is indicated within the respective circles. The values between each pair of groups denote the population divergence index (*Fst*). **(D)** Pairwise comparisons of *Fst* values between groups G1 and G2. G1 is *G. hirsutum* with the white fiber. G2 is *G. hirstum* with the brown fiber. **(E)** Pairwise comparisons of *Fst* values between G1 and G2 on chromosome A07.

To analyze the genetic variation of the distal region of chromosome A07 in cotton, we utilized resequencing data from 98 cotton accessions, including 60 *G. hirsutum* (AD1), 19 *G. arboreum* (A2), and 19 *G. barbadense* (AD2). These data were mapped to the reference genome of *G. hirsutum* (TM-1). We identified a total of 120,126 high-quality SNPs. Population structure analysis and construction of a phylogenetic tree were conducted to understand the genetic diversity within populations. The 98 accessions were classified into four subgroups (G1, G2, G3, and G4) when the value of k was set to 4 (**Figure 2A** and **Supplementary Table 4**). G1 subgroup primarily consisted of *G. hirsutum* with white fiber, G2 subgroup predominantly included *G. hirsutum* with brown fiber, G3 subgroup mainly represented individuals of *G. barbadense*, and G4 subgroup was mainly composed of *G. arboreum*. The results from the phylogenetic tree were consistent with results of population genetic structure analysis (**Figures 2A, B** and **Supplementary Table 4**).

The nucleotide diversity (π) of G1 (0.00038) and G2 (0.00049) subgroups was lower compared to G3 (0.0044) and G4 (0.00087) (**Figure 2C**). Both G1 and G2 subgroup belonged to *G. hirsutum*, while G3 and G4 subgroup were different species. By comparing the *Fst* values between each group, we observed that the *Fst* between G1 and G2 subgroup was the lowest, while the differences in *Fst* between the other two groups were more significant. These results suggest that upland cotton generally has lower genetic diversity and a more stable population. Furthermore, the data indicated that G2 subgroup had higher genetic diversity than G1 subgroup, indicating that brown-fibered upland cotton maintains greater genetic diversity compared to white-fibered upland cotton. Additionally, the results revealed that prolonged artificial selection for long-fibered upland cotton varieties has gradually reduced fiber color diversity, resulting in decreased genetic diversity in modern cultivated white-fibered upland cotton.

Although the *Fst* difference between G1 and G2 subgroup is relatively low (**Figure 2C**), we still observed some distinct variations in the genome-wide comparison. Notably, there is a significant peak on chromosome A07 (**Figure 2D**). Subsequently, we conducted an analysis specifically focusing on the chromosome A07 and found a distinct difference in the distal region of chromosome A07 (**Figure 2E**). This observation suggests that distal region of chromosome A07 may play a crucial role in determining the brown fibers in cotton. These findings align with previous research that has identified genes responsible for controlling cotton fiber color on the chromosome A07 (Wen et al., 2018).

### Analysis of the GST gene family copy number and expression in the distal region of cotton chromosome A07: implications for anthocyanin transport

**Figure 3:**
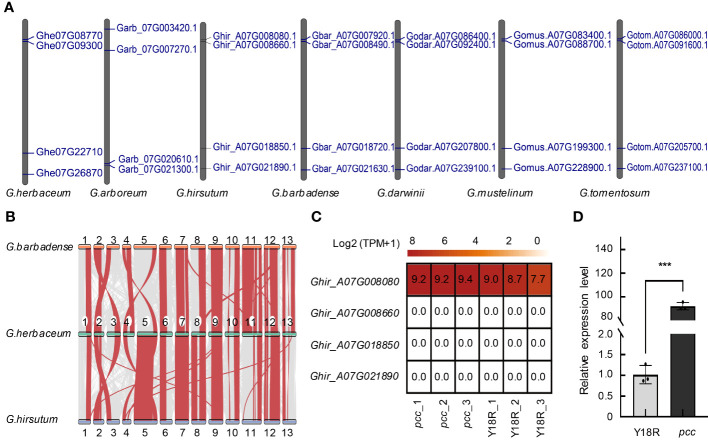
The distribution, Synteny, and expression of the GST gene. **(A)** The distribution of GST gene family members across chromosome A07 or 07 in seven cotton varieties. **(B)** The synteny analysis of GST genes among *G. herbaceum*, *G. hirsutum*, and *G. barbadense*. **(C)** The expression patterns of GST genes located on chromosome A07 during the flowering stages. The analysis is based on data from three biological replicates for each group. **(D)** Validation of *Ghir_A07G008080* expression in Y18R and *pcc*. Data are presented as mean ± SD from three replicates. Statistical significance is denoted by asterisks: * *p*<0.05; ** *p*<0.01; *** *p*<0.001, determined by t-test.

Gene mapping was conducted on GST genes in 8 different cotton varieties, namely, *G. herbaceum* (A1), *G. arboretum* (A2), *G. raimondii* (D5), *G. hirsutum* (AD1), *G. barbadense* (AD2), *G.mustelinum* (AD3), *G. tomentosum* (AD4), *G. darwinii* (AD5), revealing their distribution across all chromosomes in different cotton species. We identified 70, 59, 73, 110, 113, 134, 157, and 149 GST genes in these cotton varieties, respectively. Interestingly, the number of GST gene family members in tetraploid cotton is approximately twice that in diploid cotton (**Supplementary Figure 2A**), it reveals that genome duplication results in a twofold increase in the number of genes. Notably, chromosome Chr03 exhibited the highest number of tandemly duplicated gene clusters for GST genes, while chromosome 06 had the fewest in all diploid cotton species (**Supplementary Figures 3**, **4**). However, in tetraploid cotton, a significant number of GST genes were found on chromosome A02 and D02, with some forming tandemly duplicated gene clusters (**Supplementary Figures 5**-**9**). In contrast, chromosome A07 contained only four GST genes in both diploid and tetraploid cotton species, and no gene clusters were observed resulting from tandem duplication of GST genes (**Figure 3A**). The number and location of GST genes on chromosome A07 are consistent throughout the evolution of cotton from diploid to tetraploid, indicating that the gene count remains unchanged during the whole genome duplication (WGD) and tandem duplication events. This result emphasizes the conservative nature of GST genes on the chromosome A07.

Subsequent synteny analysis of the GST gene family revealed a high collinearity of GST genes on chromosome A07 in *G. herbaceum*, *G. hirsutum*, and *G. barbadense*, suggesting their likely origin from *G. herbaceum* (**Supplementary Figure 19**). In *G. hirsutum* and *G. barbadense*, homologous GST genes on chromosomes A02 and A03 exhibited positional changes compared to *G. herbaceum*, indicating a replacement of chromosomes A02 and A03 in the A subgenome during the diploid to tetraploid transition (**Figure 3B**). Furthermore, synteny analysis between tetraploid cotton species and *G. arboreum* with *G. herbaceum* revealed a higher homology of the A subgenome of all tetraploid cotton species with *G. herbaceum* (**Supplementary Figure 19**).

Observations from the constructed phylogenetic tree reveal that *Ghe07G08770*, *Garb_07G021300*, *Ghir_A07G008080*, *Gbar_A07G007920*, *Godar.A07G086400*, *Gotom.A07G086000*, *Gomus.A07G083400* cluster together in the Phi subfamily (**Supplementary Figure 21** and **Supplementary Table 5**). Previous studies have indicated that plant GST genes in the Phi subfamily are involved in the transport and accumulation of flavonoids (Kitamura et al., 2012). Sequence alignments demonstrate a high degree of conservation in their sequences, with only two mutation sites identified in *G. herbaceum* (**Supplementary Figure 22**), further supporting the notion of high conservation of GST genes related to anthocyanin accumulation and transport across different cotton species. Real-time PCR and RNA-seq analysis to investigate the expression patterns of GST genes in the *pcc* and Y18R of *G. hirsutum* during flowering time, reveal that the expression of only the *Ghir_A07G008080* (*PCC*) gene on the chromosome A07 is detected in flowers. Its expression in *pcc* is significantly higher than in the control cotton Y18R, reaching 90 times the expression level of Y18R (**Figures 3C, D** and **Supplementary Figure 17**) The expression of the other three genes is barely detectable (**Supplementary Figure 17C**). It further confirms the key gene of *Ghir_A07G008080* influencing the formation of different flower colors in *G. hirsutum*.

### Exploring the MYB TF family in the distal region of cotton chromosome A07: implications for the anthocyanin pathway

To gain insights into the regulation of reproductive organ color by MYB transcription factors located on the distal end of chromosome A07 in cotton, we conducted a comprehensive analysis. Firstly, we identified the MYB genes present in various cotton species. We respectively identified 216, 211, 218, 416, 426, 430, 438, and 429 MYB genes in the eight cotton varieties (**Supplementary Figure 2B**). Subsequently, we plotted their genomic locations on a chromosomal map, providing a visual representation of their distribution. The distribution of MYB genes across chromosomes in different cotton species was found to be random and uneven. Specifically, in diploid cotton, the MYB genes were predominantly located on chromosomes 05, 07, 08, 11, 12, and 13. Similarly, in tetraploid cotton, the majority of MYB genes in the A subgenome were found on chromosomes A05, A07, A08, A11, A12, and A13 (**Supplementary Figures 10**-**16**).

**Figure 4:**
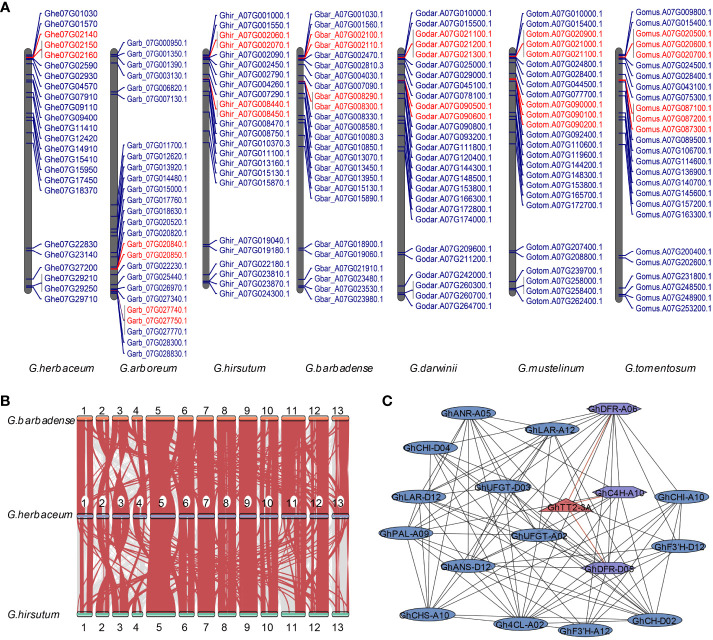
Examination of the MYB gene family located on Chromosome A07. **(A)** The localization of the MYB gene family on chromosome 7 of seven cotton varieties in A subgeome. Genes highlighted in red indicate those that have arisen through tandem duplication events. **(B)** The synteny analysis of MYB genes among *G. herbaceum*, *G. hirsutum*, and *G. barbadense*. **(C)** The protein interaction analysis focuses on MYB genes located on chromosome A07 and their interactions with proteins involved in pigment synthesis. MYB genes on chromosome A07 are represented by red triangles, while the interacting proteins are shown as purple rectangles. The correspondence between gene name and gene ID can be viewed in **Supplementary Table 6**.

We made some interesting observations in our study. Firstly, we found tandem repeat gene clusters on both chromosome 07 in diploid cotton and A07 in tetraploid cotton (**Figure 4A**), except in *G. herbaceum*, where only one tandem repeat gene cluster was present on chromosome A07. Secondly, during synteny analysis of the MYB gene family, we discovered a high degree of homology between chromosome 07 in *G. herbaceum* and the A07 chromosome in *G. hirsutum* and *G. barbadense* (**Figure 4B**). Next, we observed the expression levels of MYB genes on the A07 chromosome in white and brown cotton at 0, 5, 10, 15, and 20 days post-anthesis (DPA). It was evident that only the *Ghir_A07G002090* gene showed significant differences between white and brown cotton fibers. The differences were most pronounced at 5, 10, and 15 DPA, reaching approximately 2-3 times higher expression levels in white cotton fibers (**Supplementary Figure 18**). Lastly, the synteny analysis revealed that the A07 chromosome remained evolutionarily conserved during the transition from diploid to tetraploid, showing no significant variation (**Supplementary Figure 20**). This conservation is likely attributed to the importance of maintaining stability in the anthocyanin metabolism process, thereby preventing substantial changes.

Our protein-protein interaction analysis focused on the interactions between MYB proteins on the chromosome A07 and proteins involved in the anthocyanin and proanthocyanin pathways. For example, we observed that *GhTT2-3A* showed interactions with proteins related to anthocyanin synthesis, such as GhDFR_A06, GhC4H_A10 and GhDFR_D05. (**Figure 4C** and **Supplementary Table 6**). This finding further suggests that MYB TF in the distal region of cotton chromosome A07 plays a crucial role in the anthocyanin metabolism process and likely influences the coloration of brown cotton fibers.

## Discussion

In this study, we pinpointed two MYB transcription factor genes, *GbBM* and *GhTT2-3A*, which are pivotal in anthocyanin synthesis, influencing the purple spots at petal bases and the brown hue of fibers, respectively. Alongside, we identified the *PCC/GhTT19* gene, crucial for anthocyanin transport to vacuoles, thereby dictating petal redness, located in the distal region of chromosome A07. These genes collectively form a cluster that orchestrates the coloration in cotton’s reproductive structures. Our population genetics analysis revealed that significant genetic variability in chromosome A07’s distal region plays a crucial role in determining fiber brown coloration. Moreover, the pronounced conservation observed at this chromosome’s distal end underscores its role in ensuring anthocyanin metabolic stability, potentially boosting offspring’s reproductive success.

The finding that non-homologous genes with similar functions can form metabolic gene clusters highlights a key evolutionary process, distinct from bacteria’s horizontal gene transfer (Richard et al., 2008; Sue et al., 2011; Takos et al., 2011). In eukaryotes, these clusters likely result from gene duplication, neofunctionalization, and dynamic genomic rearrangements, a departure from bacterial operon structures where genes are co-transcribed. Eukaryotic cluster genes are transcribed separately, indicating a sophisticated regulatory system (Nutzmann et al., 2018). Whole-genome duplication (WGD) is a critical evolutionary milestone, leading to rapid diploidization as redundant genes are eliminated to preserve genomic stability, though some duplicates remain (Freeling, 2009; Li et al., 2021). These duplications are fundamental to evolutionary development.

Plant gene clusters can arise through tandem repeats, as seen in the maize TPS gene cluster, or through dispersed repeats facilitated by transposons, like in the poppy (Winzer et al., 2012; Ding et al., 2020). These clusters typically locate in chromosomal hotspots such as telomeres and centromeres, areas prone to recombination, rearrangement, and translocation, promoting gene cluster diversification and formation (Field et al., 2011; Field and Osbourn, 2012; Zhou et al., 2022). Yet, the regulatory mechanisms governing plant metabolic gene clusters remain largely elusive, with scant examples at the transcriptional and chromatin levels. For instance, in Oryza sativa, the OsTGAP1 transcription factor, triggered by chitin oligosaccharides, controls a diterpenes gene cluster, influencing diterpene production (Okada et al., 2009). Similarly, in *Arabidopsis thaliana*, a triterpene gene cluster undergoes spatial conformational changes in its structural domains upon activation or suppression, underscoring a sophisticated regulatory framework (Nutzmann et al., 2020).

Reproductive organ coloration is vital for attracting pollinators and enhancing plant resilience, driven by the evolutionary interaction between plants and pollinators (Zhu et al., 2017; Su et al., 2020). This interaction has led to the development of flower colors and patterns that maximize visibility and attractiveness according to pollinator preferences (Chittka and Raine, 2006). For example, *G. hirsutum*’s yellow petals with purple spots not only provide UV protection but also enhance visibility for pollinators, and its yellow petals are particularly attractive to bees (Abid et al., 2023). In contrast, modern upland cotton has developed milky-white, unspotted flowers, likely due to selective breeding aimed at minimizing dependence on insect pollinators, leading to a gradual loss of distinctive coloration and patterns (Zhang et al., 2016; Cai et al., 2023). This coloration is closely associated with anthocyanin metabolism, essential for the plant’s anti-aging, antioxidant, and stress resistance capabilities (He and Giusti, 2010; Alappat and Alappat, 2020). The coloring process is regulated by transcription factors that control anthocyanin biosynthesis genes and transport proteins that facilitate pigment accumulation (Marrs et al., 1995; Quattrocchio et al., 1999; Sun et al., 2012; Ni et al., 2023). Our research indicates that the red petals of *G. hirsutum pcc* and the purple spots on *G. barbadense* petals result from anthocyanin accumulation, triggered by specific gene activity. Additionally, the brown fiber coloration in cotton is due to the upregulated expression of the *GhTT2-3A* gene, which boosts proanthocyanidin biosynthesis and accumulation, key to the plant’s pigment metabolism.

We have shown that *GhTT2-3A*, *GbBM*, and *PCC*/*GhTT19* form a cluster at the distal end of chromosome A07. This arrangement implies a synergistic effect on the metabolic processes of anthocyanin and proanthocyanidin. The location of these genes near the telomeric region suggests that gene rearrangements and exchanges during population differentiation might have played a role in bringing together genes that regulate both anthocyanin and flavonoid biosynthesis and transport within this specific genomic area. This clustering is believed to boost gene functionality, facilitate regulatory interactions among the genes, and enhance the efficiency of enzymatic reactions within the cluster (Rocha, 2008; Shen et al., 2021). Moreover, this part of the genome shows high stability in tetraploid cotton, indicating that the initial grouping of these genes was vital for preserving the stability of anthocyanin and pigment metabolism. These findings provide valuable insights into the genetic mechanisms behind color variation in cotton reproductive organs, deepening our understanding of the complex regulatory networks that control pigment biosynthesis.

## Data availability statement

The original contributions presented in the study are included in the article/**Supplementary Material**. Further inquiries can be directed to the corresponding author.

## Author contributions

LZ: Writing – original draft, Writing – review & editing, Software, Methodology. JZ: Methodology, Software, Writing – review & editing. HH: Methodology, Writing – review & editing. ZM: Methodology, Writing – review & editing. YW: Data curation, Investigation, Resources, Writing – review & editing. SG: Data curation, Methodology, Writing – review & editing. CL: Supervision, Writing – original draft, Writing – review & editing, Conceptualization, Project administration.
