# Supplementary material for: Anthocyanin gene enrichment in the distal region of cotton chromosome A07: mechanisms of reproductive organ coloration
Source: Front Plant Sci. 2024 Apr 18;15:1381071. doi: 10.3389/fpls.2024.1381071 (PMC11063239; doi:10.3389/fpls.2024.1381071)
Supplement: Supplementary file 1 [file DataSheet_1.pdf]

Supplementary Material

Anthocyanin gene enrichment in the distal region of cotton chromosome A07: mechanisms of reproductive organ coloration

Liuchang Zheng[1,2,#], Jilong Zhang[2,#], Haiyan He[2,#], Zhigang Meng[2,#], Yuan Wang[2], Sandui Guo[2], and Chengzhen Liang[2,*]

* **Correspondence:** Chengzhen Liang: liangchengzhen@caas.cn

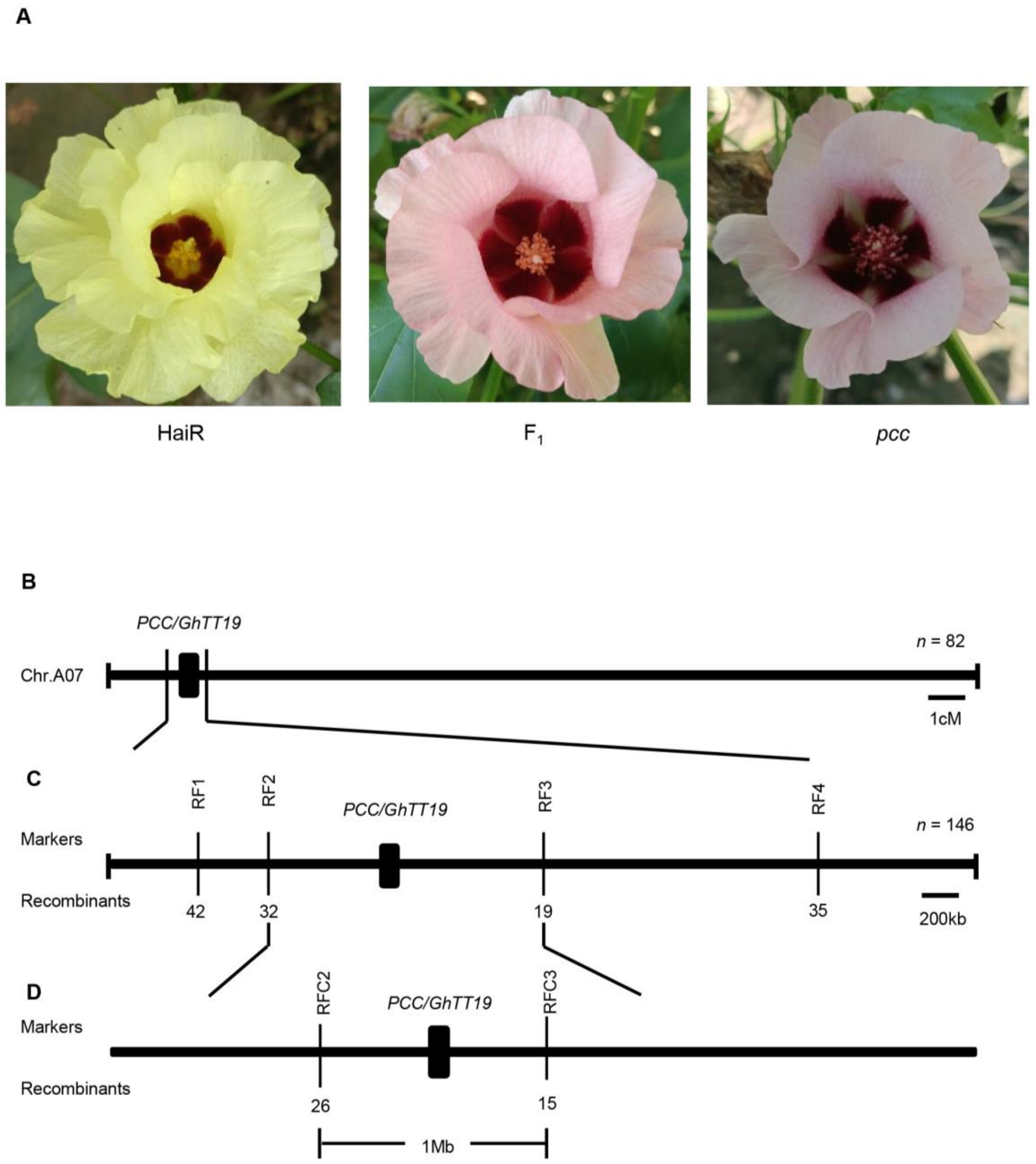

Supplementary Figure 1 Cloning of the *PCC/GhTT19* gene. (A) The flower color of phenotypes of the HaiR, *pcc* and F_1 (HaiR×*pcc*) during the flowering time. (B) Location of *PCC/GhTT19*on *G. hirsutum* chromosome A07. (C) Coarse linkage map of *PCC/GhTT19*. The number of recombinants in the F_2 population derived by HaiR and *pcc* between the marker and *PCC/GhTT19* is indicated. (D) High-resolution linkage map of *PCC/GhTT19*.

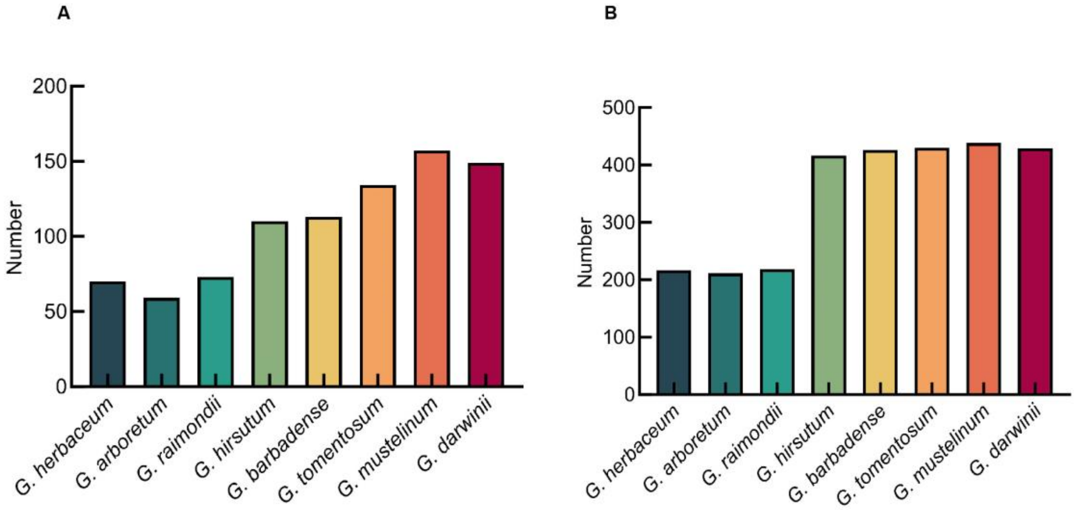

Supplementary Figure 2 Identification of GST and MYB genes in eight cotton varieties.

(A)The number of GST genes. (B) The number of MYB genes.

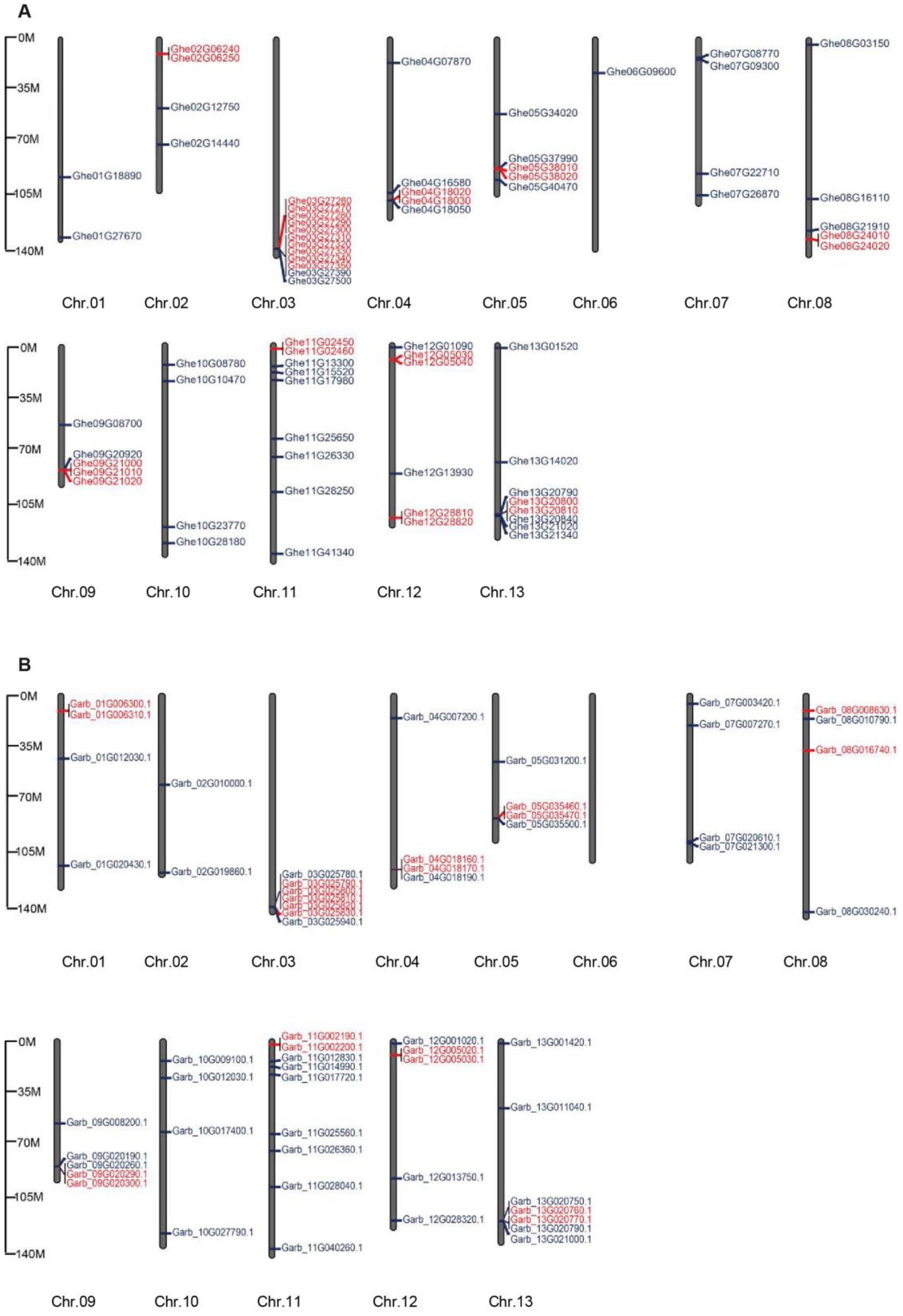

Supplementary Figure 3 Localization of the GST gene family in *G. herbaceum* and *G. arboretum*. (A) The localization of the GST Gene family in *G. herbaceum.* (B) The localization of the GST Gene family in *G. arboretum*. The genes highlighted in red are generated by tandem duplication.

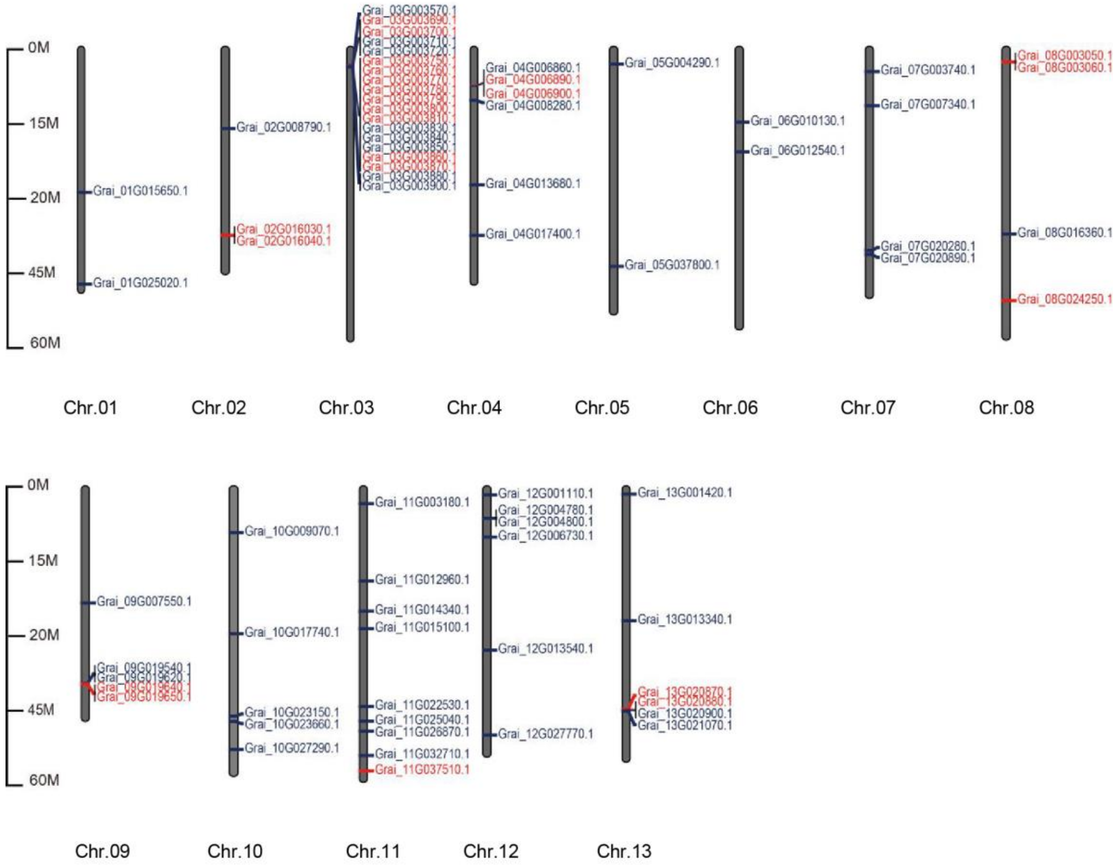

Supplementary Figure 4 Localization of the GST gene family in *G. raimondii*.

The genes highlighted in red are generated by tandem duplication.

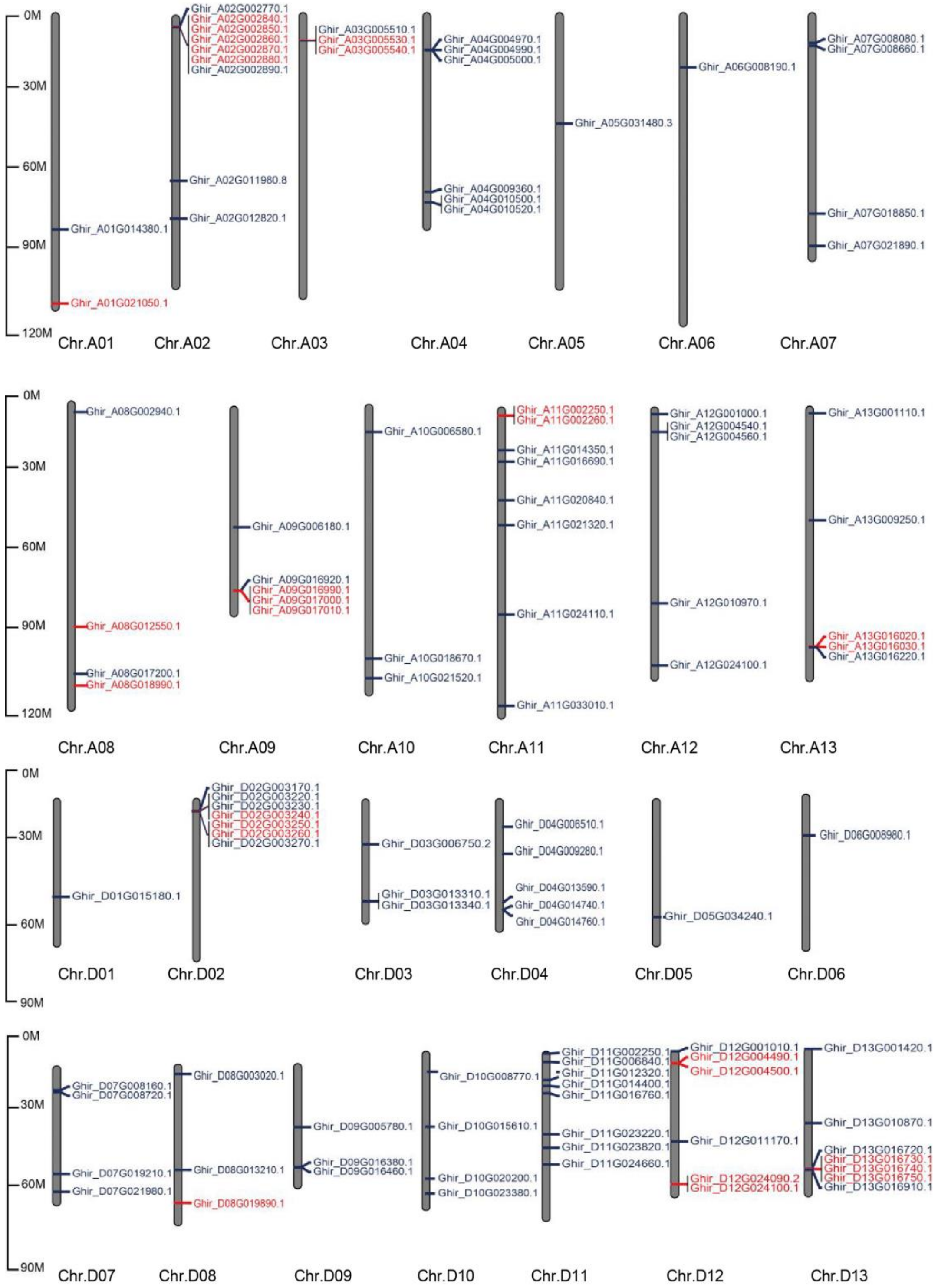

Supplementary Figure 5 Localization of the GST gene family in *G. hirsutum.*

The genes highlighted in red are generated by tandem duplication.

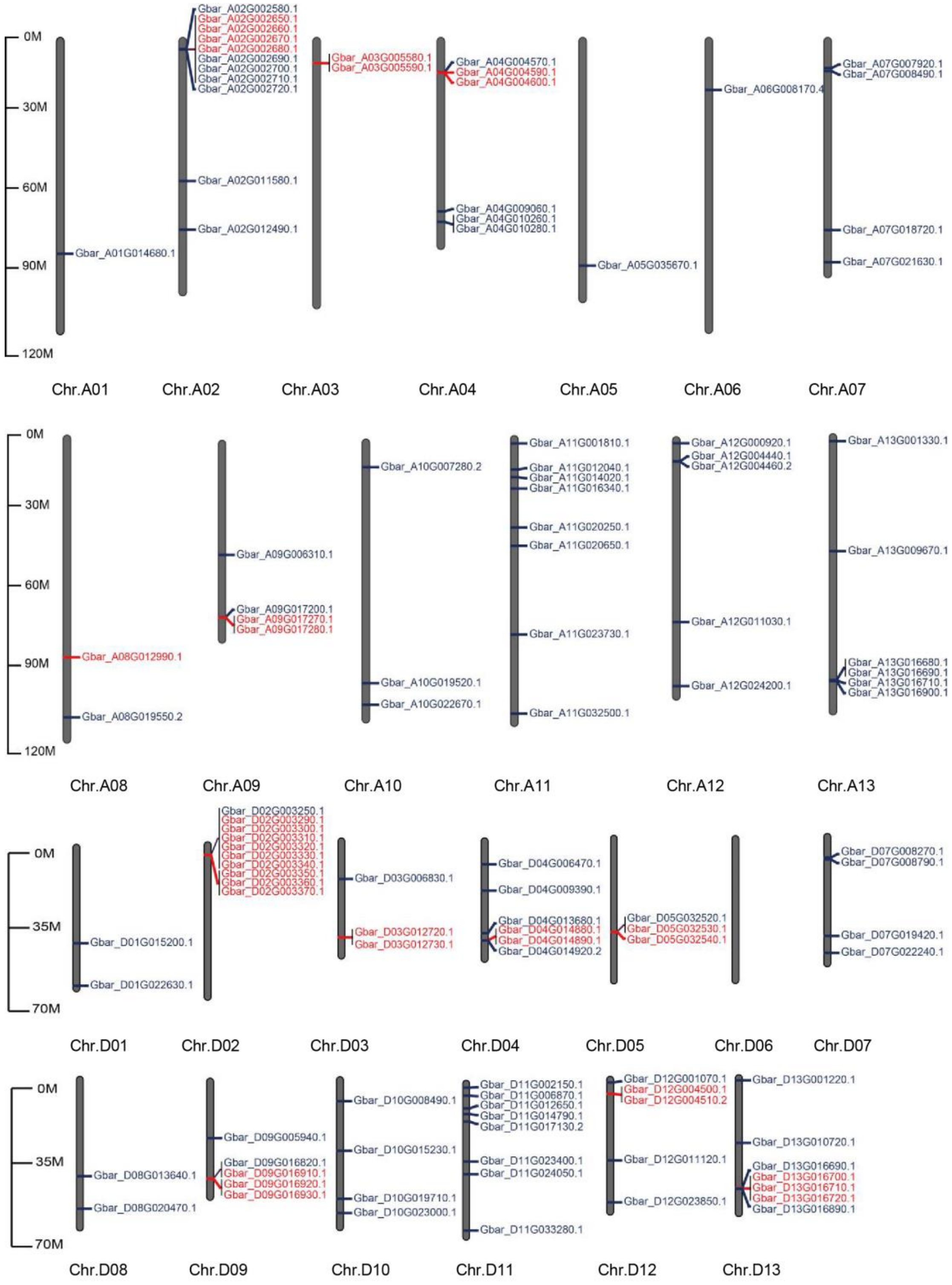

Supplementary Figure 6 Localization of the GST gene family in *G. barbadense*.
The genes highlighted in red are generated by tandem duplication.

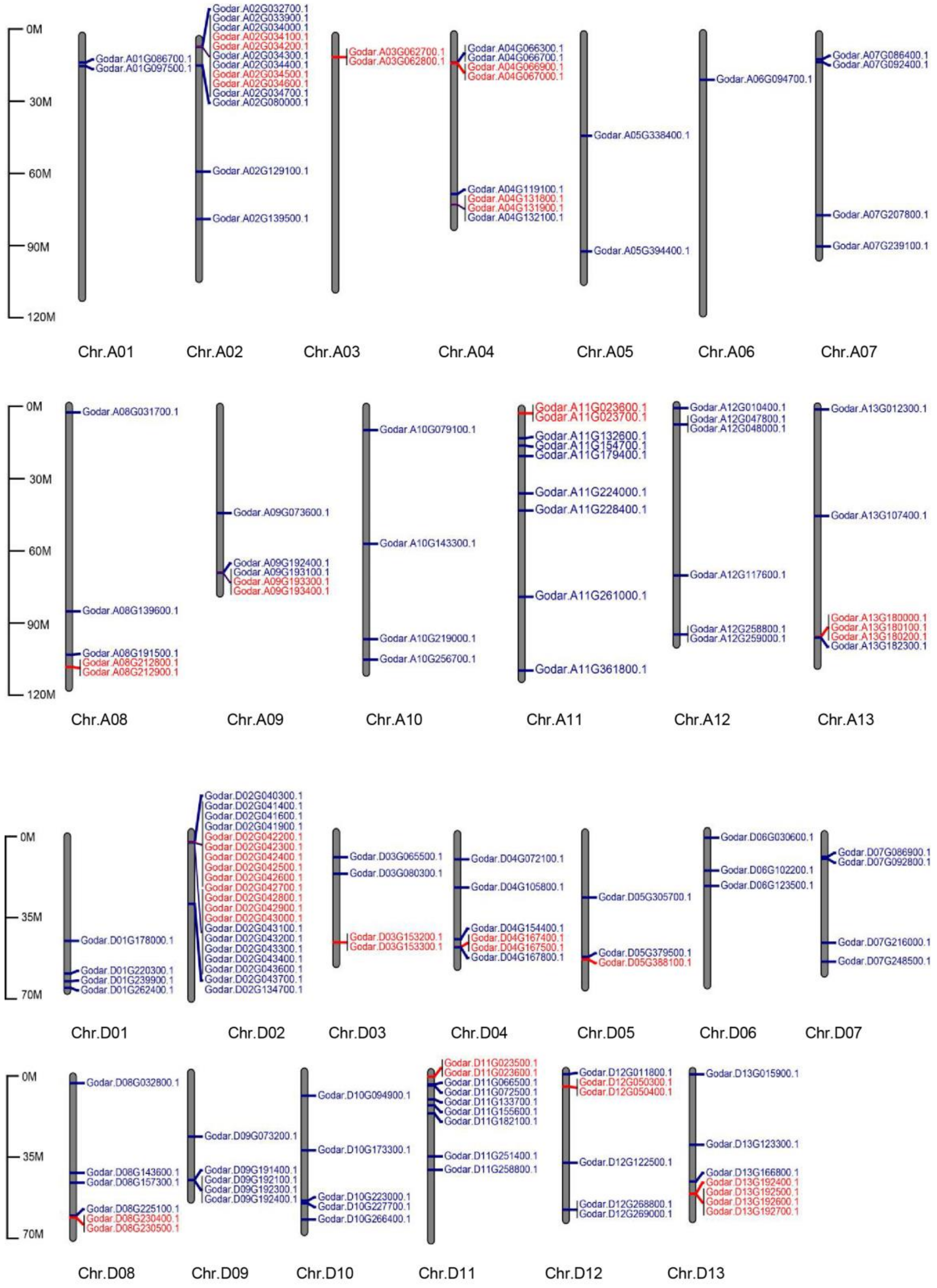

Supplementary Figure 7 Localization of the GST gene family in *G. darwinii*.
The genes highlighted in red are generated by tandem duplication.

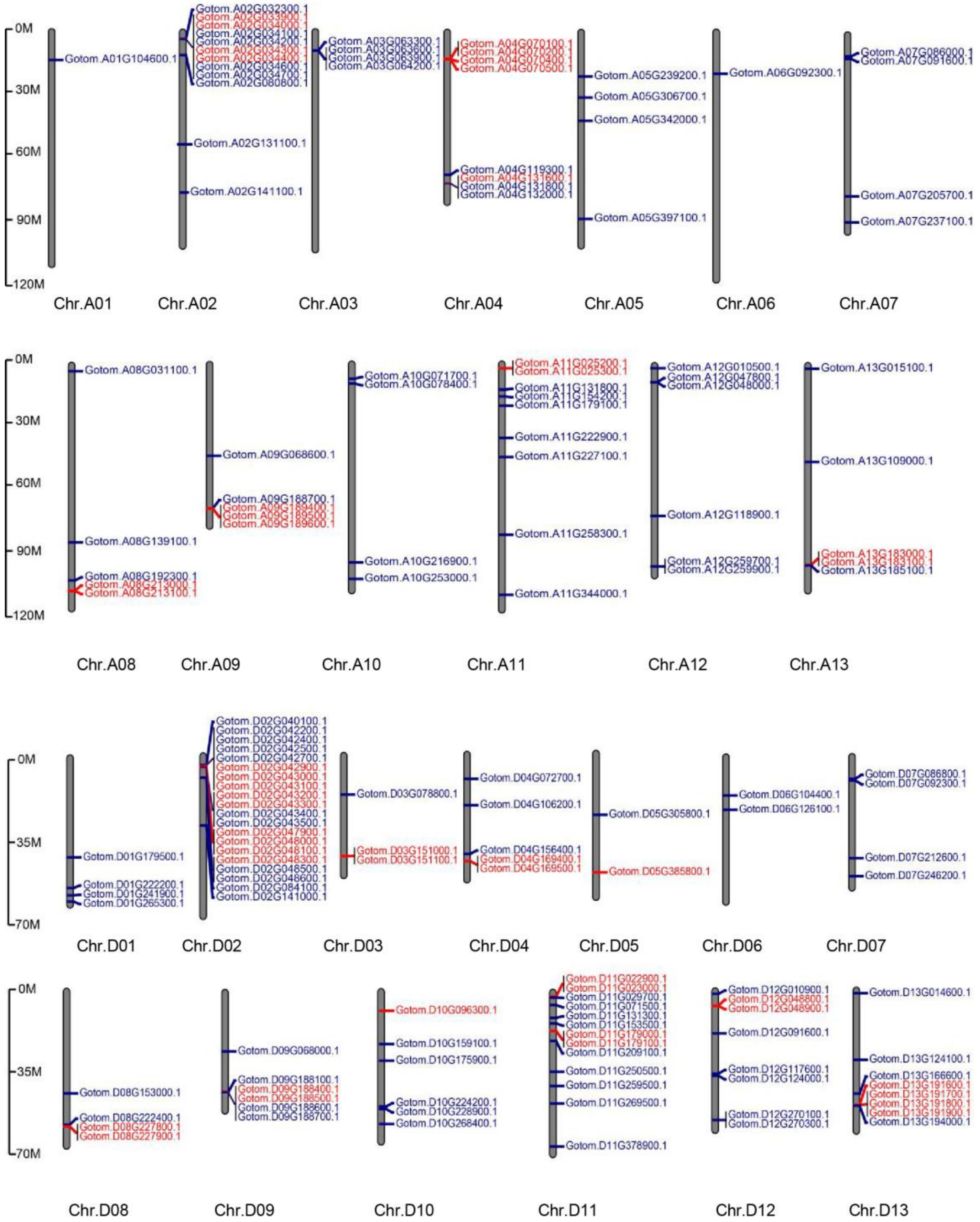

Supplementary Figure 8 Localization of the GST gene family in *G. tomentosum*.

The genes highlighted in red are generated by tandem duplication.

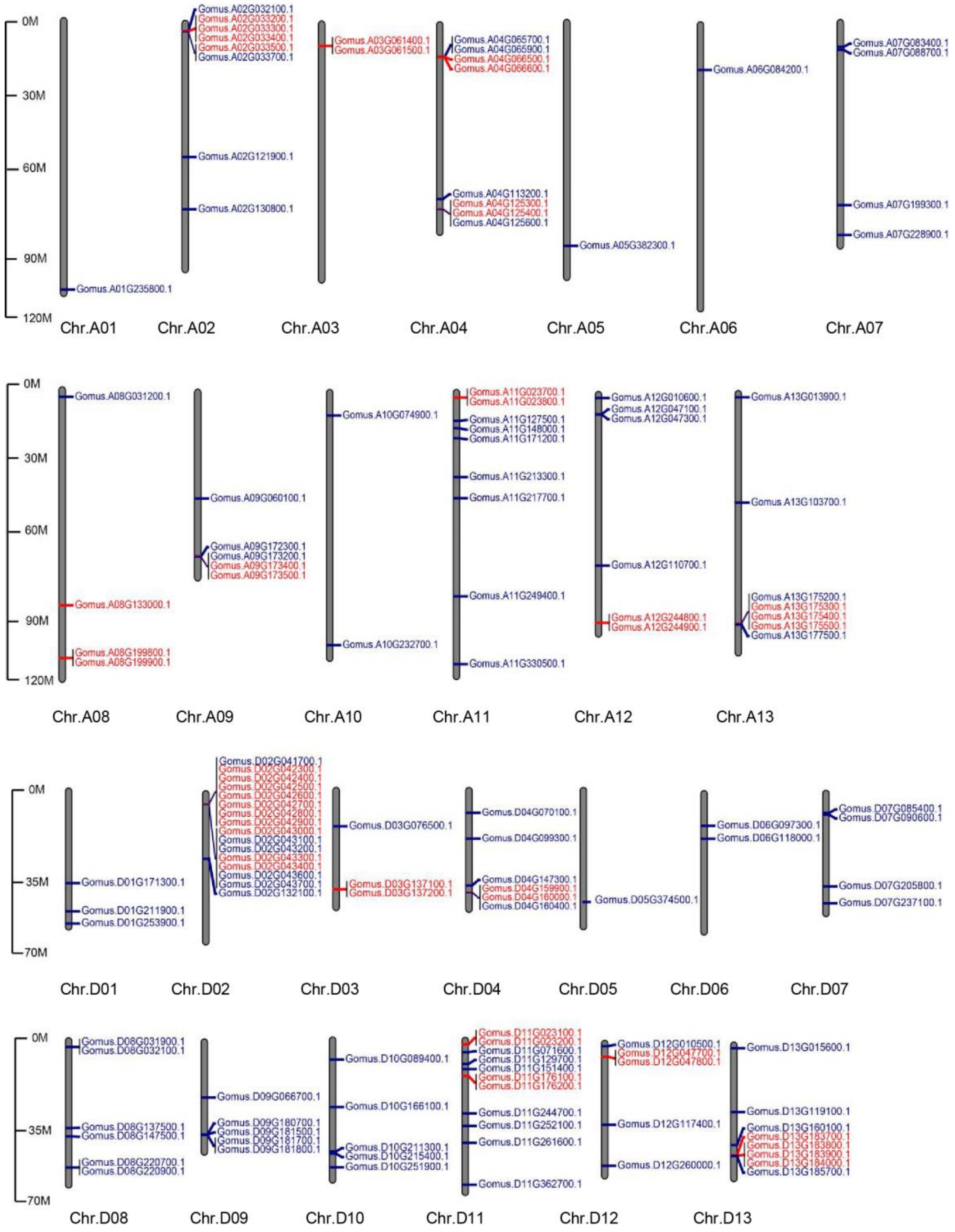

Supplementary Figure 9 Localization of the GST gene family in *G. mustelinum*.
The genes highlighted in red are generated by tandem duplication.

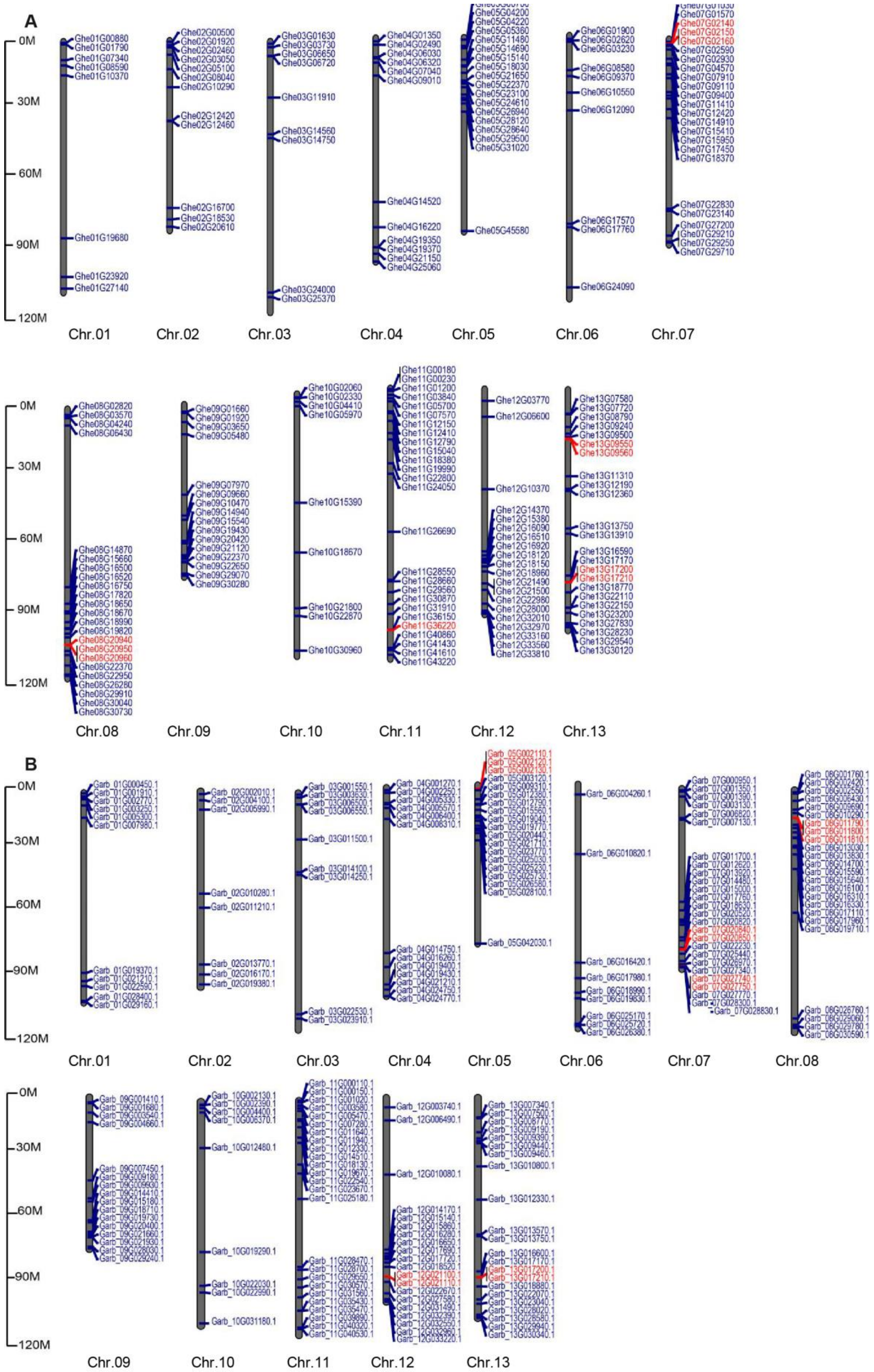
A
0M
30M
60M
90M
120M
Chr.01
Chr.02
Chr.03
Chr.04
Chr.05
Chr.06
Chr.07
Chr.08
Chr.09
Chr.10
Chr.11
Chr.12
Chr.13
B
Chr.01
Chr.02
Chr.03
Chr.04
Chr.05
Chr.06
Chr.07
Chr.08
Chr.09
Chr.10
Chr.11
Chr.12
Chr.13

Supplementary Figure 10 Localization of the MYB gene family in *G. herbaceum* and *G. arboretum*. (A) The localization of the MYB Gene family in *G. herbaceum*. (B) The localization of the MYB Gene family in *G. arboretum*. The genes highlighted in red are generated by tandem duplication.

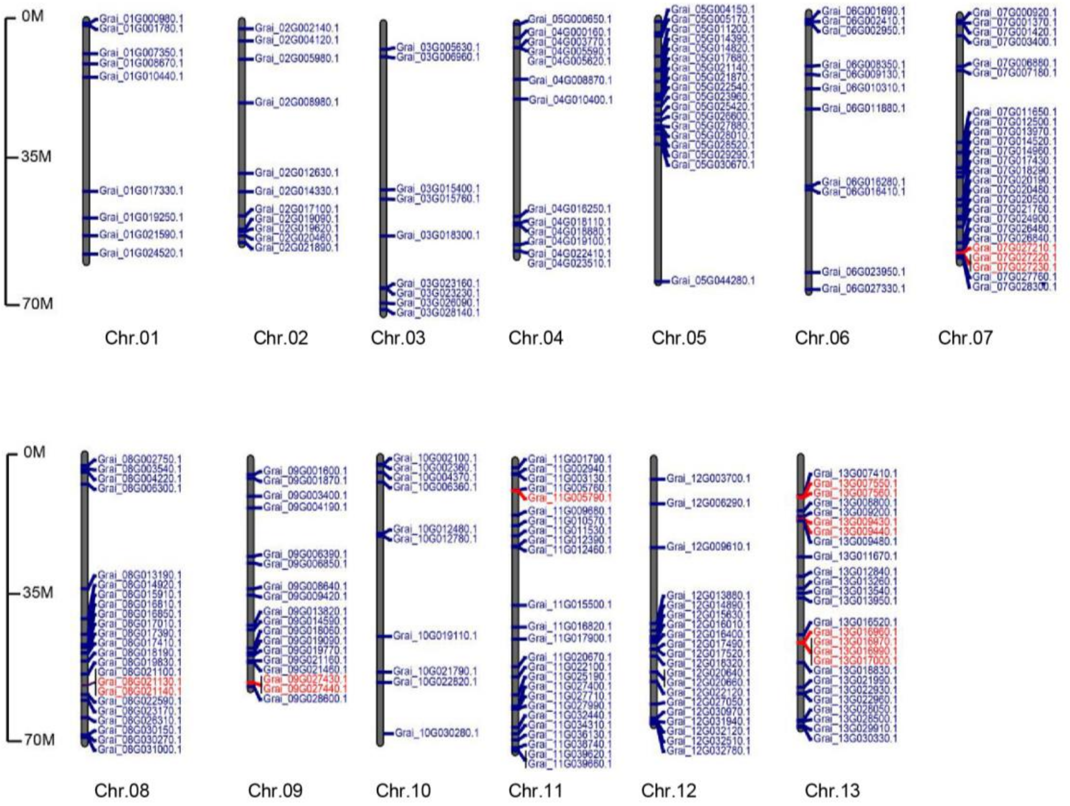

Supplementary Figure 11 Localization of the MYB gene family in *G. raimondii*.
The genes highlighted in red are generated by tandem duplication.

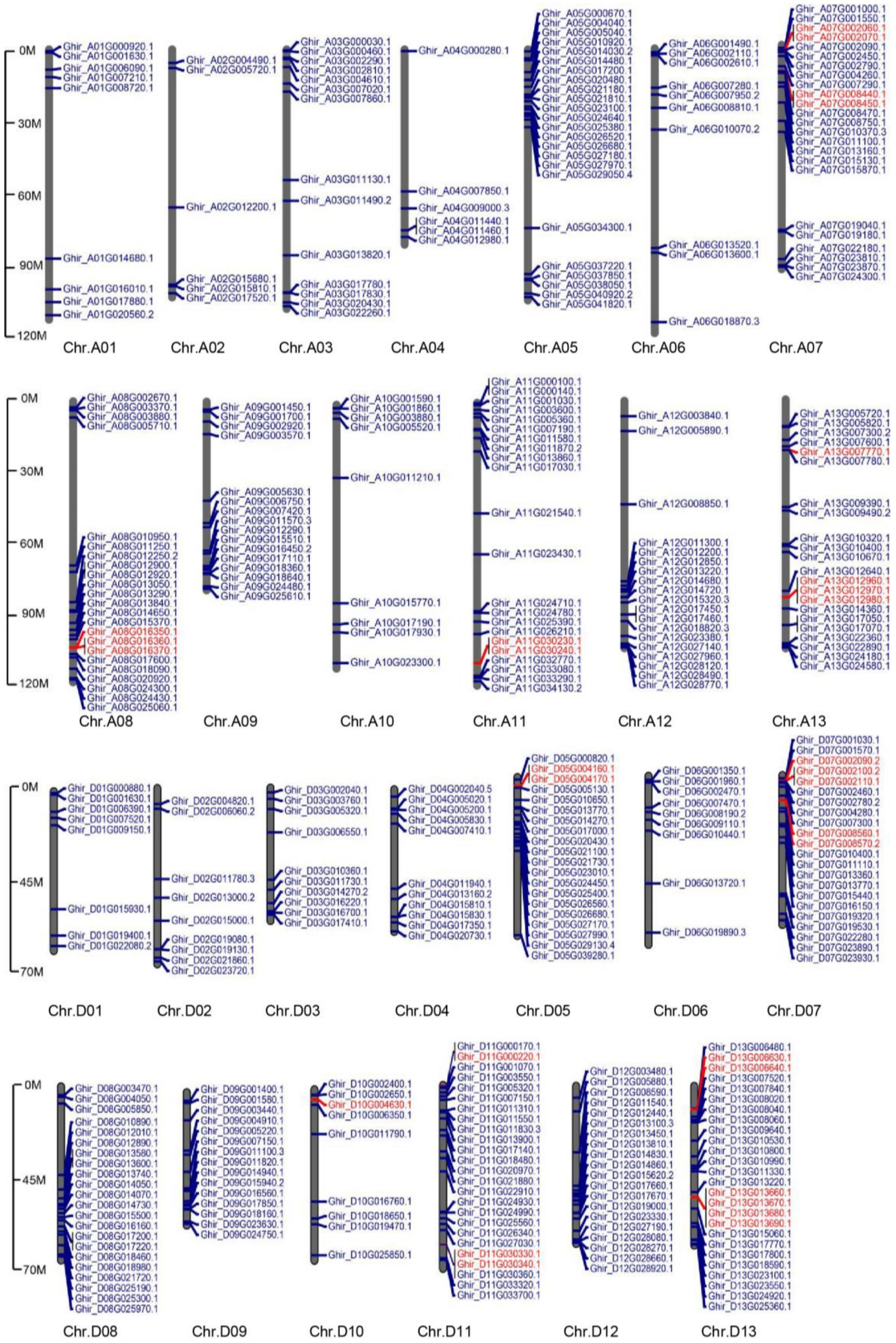

Supplementary Figure 12 Localization of the MYB gene family in *G. hirsutum*.

The genes highlighted in red are generated by tandem duplication.

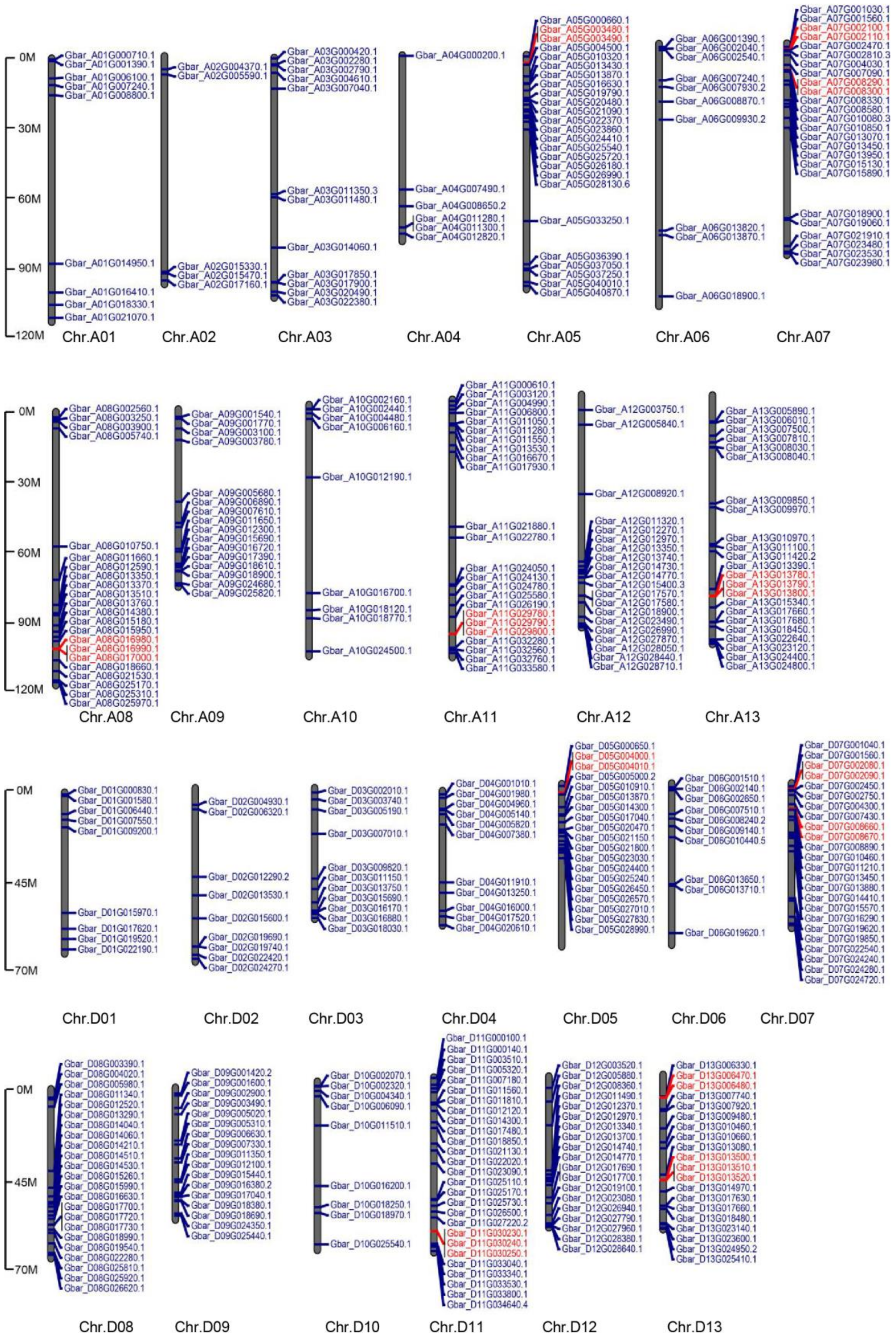

Supplementary Figure 13 Localization of the MYB gene family in *G. barbadense*.

The genes highlighted in red are generated by tandem duplication.

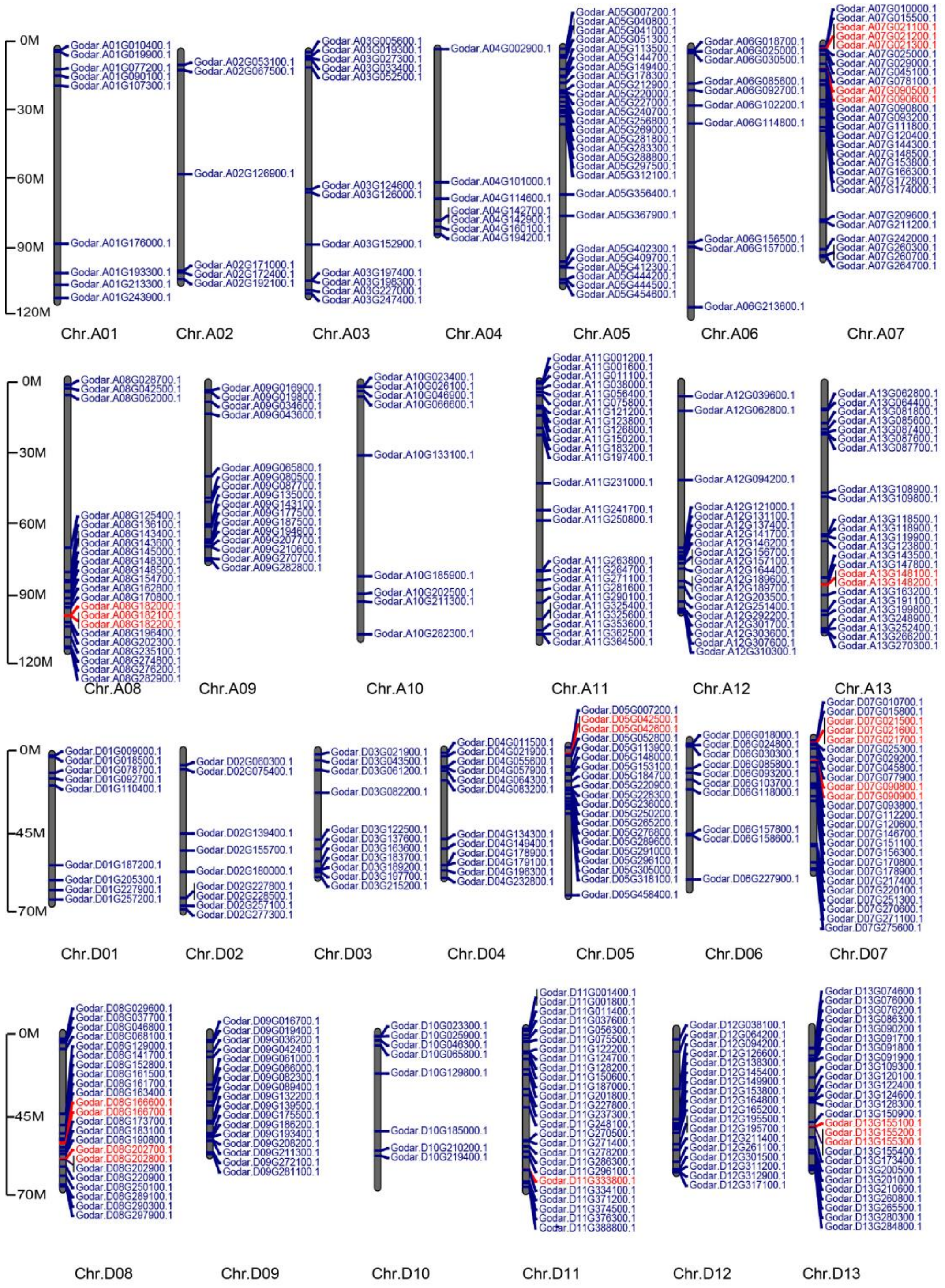

Supplementary Figure 14 Localization of the MYB gene family in *G. darwinii*.

The genes highlighted in red are generated by tandem duplication.

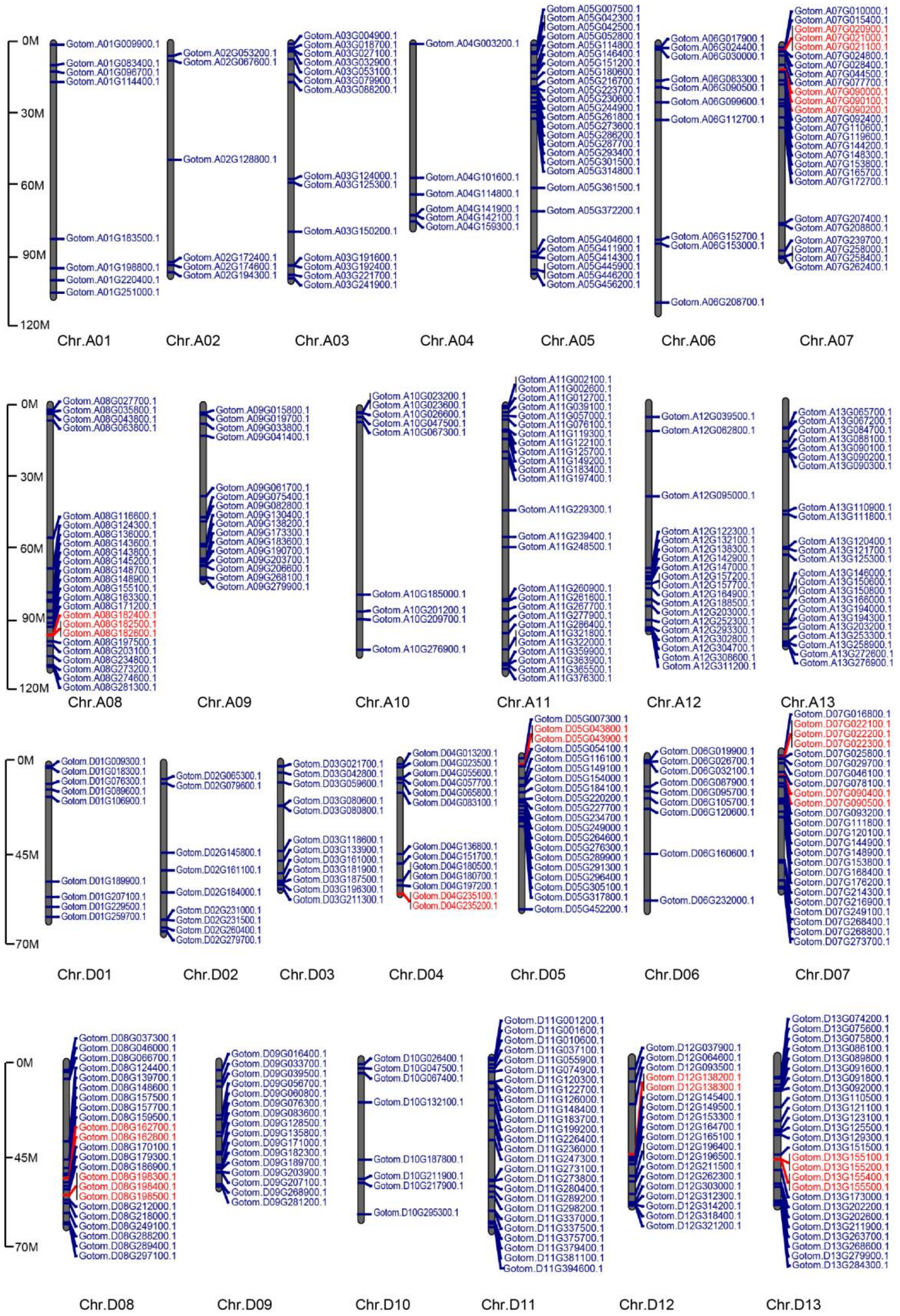

Supplementary Figure 15 Localization of the MYB gene family in *G. tomentosum*.

The genes highlighted in red are generated by tandem duplication.

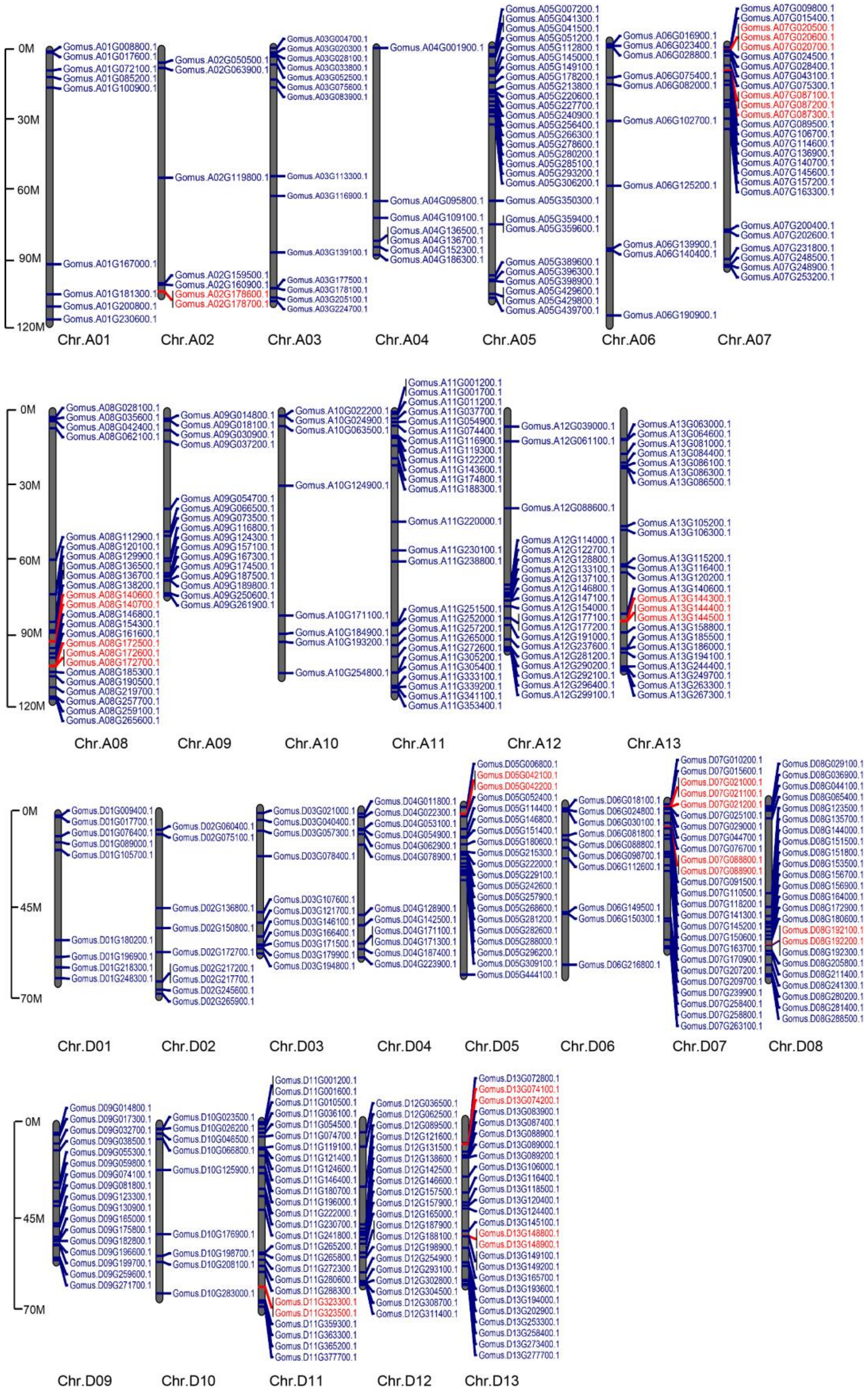

Supplementary Figure 16 Localization of the MYB gene family in *G. mustelinum*.

The genes highlighted in red are generated by tandem duplication.

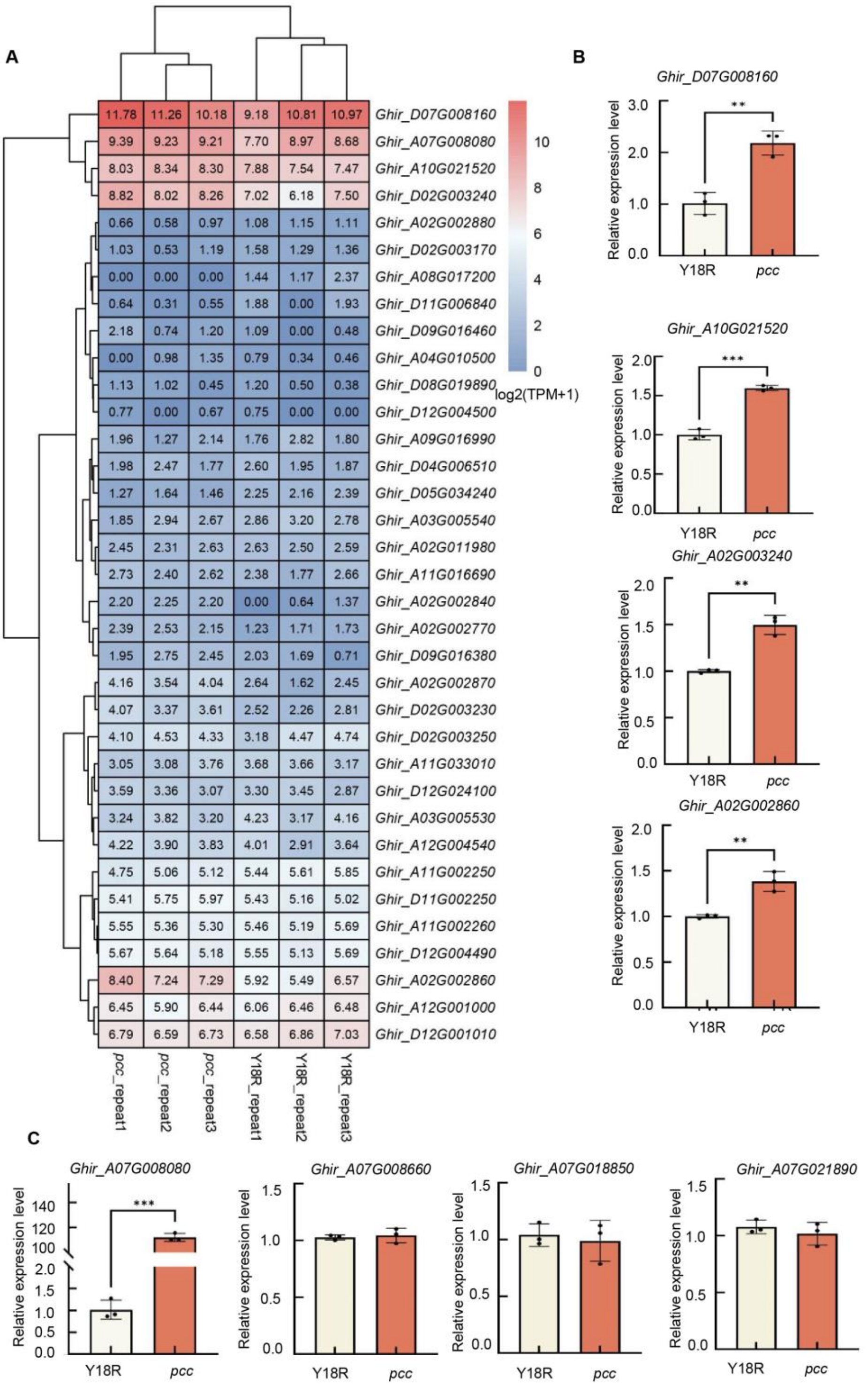

Supplement Figure 17 The gene expression analysis of GST during flowering time in *pcc* and Y18R. (A) the GST family expression analysis. (B) The qRT-PCR validation of highly expressed genes during the flowering period in *pcc*.

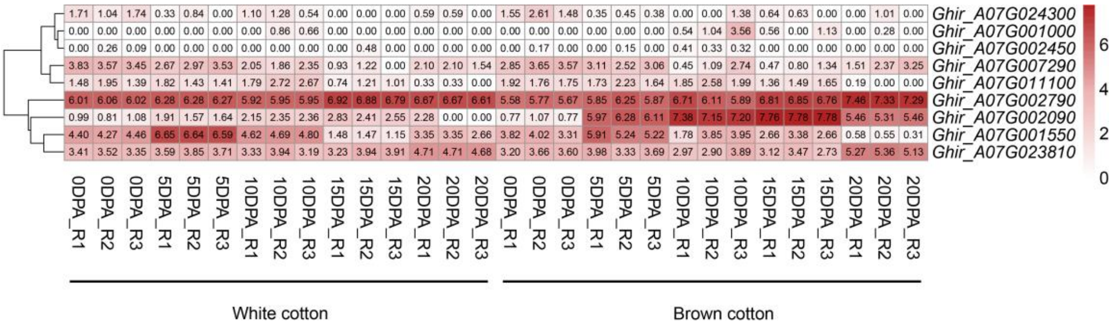

Supplementary Figure 18 The gene expression of MYB gene family of chromosome A07 in brown and white cotton at 0,5,10,15,20 DPA (days post-anthesis).

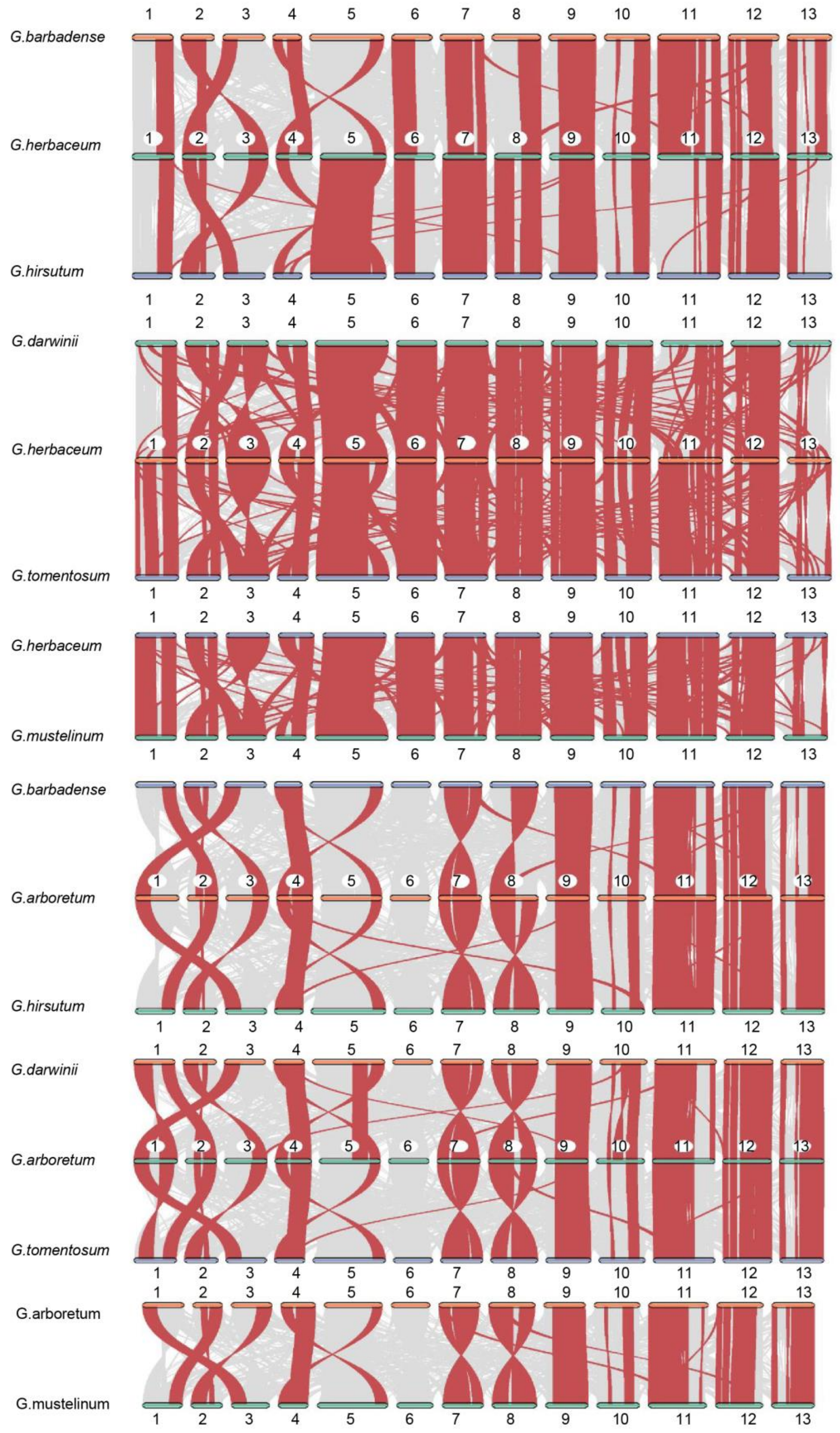

Supplementary Figure 19 Synteny analyses of the GST gene family in A subgenome of cotton.

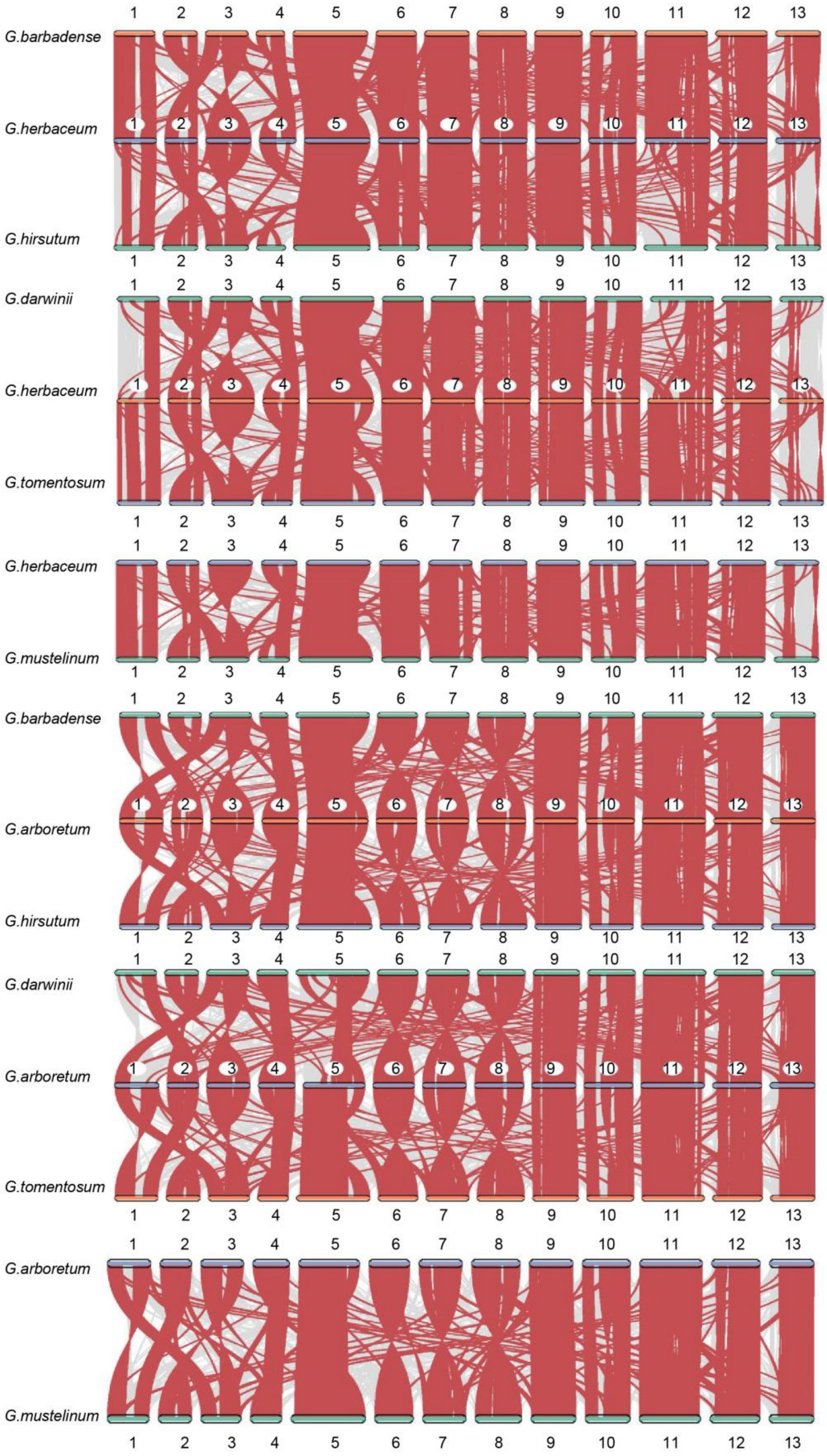

Supplementary Figure 20 Synteny analyses of the MYB gene family in A subgenome of cotton.

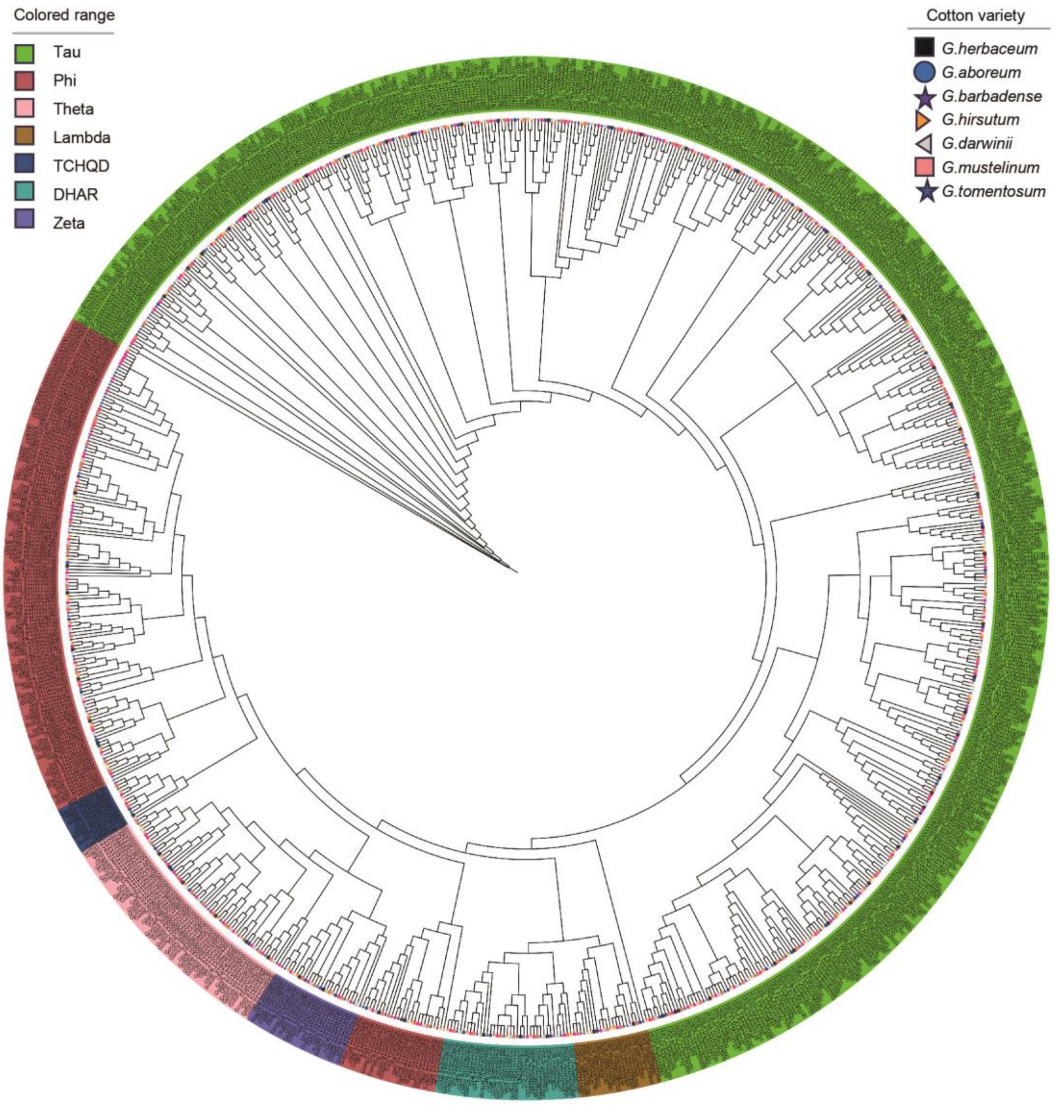

Supplement Figure 21 Phylogenetic analysis of the GST gene family derived from eight cotton varieties.

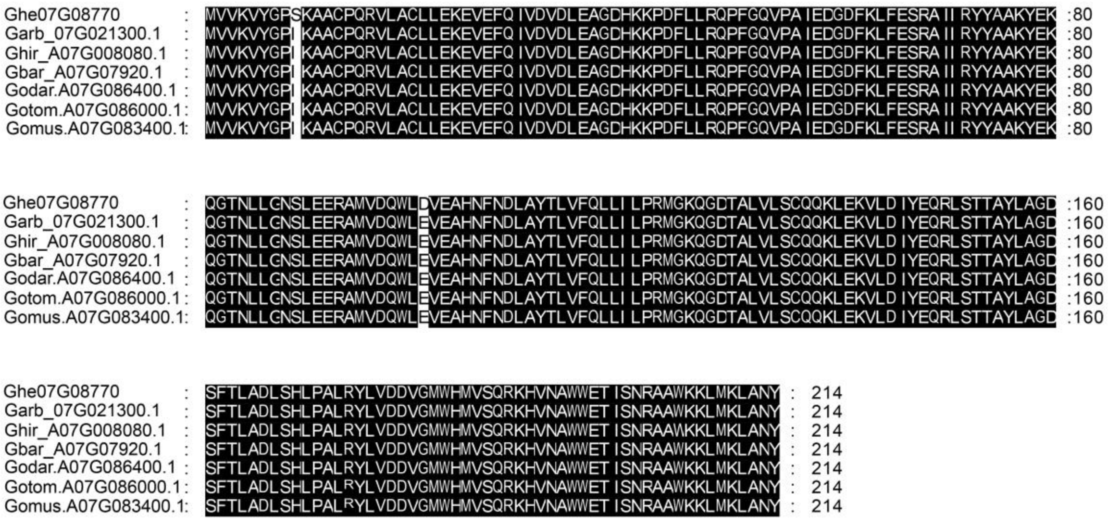

Supplementary Figure 22 Sequence alignment of proteins associated with anthocyanins located on chromosome 07 or A07 in seven cotton varieties.

Supplementary Table 1 Genetic analysis using the F_2 population derived with *G. hirsutum* line *pcc* and *G. barbadense* line HaiR. The observed ratios were tested for deviation from the expected values with a Chi-Square test for goodness-of-fit ($P < 0.05$).

Genetic population	Flower color	Number	$\chi2$
F_2	Red	599	$2.00<\chi2_{0.05}3.48$
	Yellow	270	

Supplementary Table 2 The information of genes located on chromosome A07 about cotton's reproductive organs color.

Gene ID	Gene Name	Description	Reference genome	Location(bp)
Ghir_A07G002090	*GhTT2-3A*	Transcription factor TT2	*G.hirsutum* (https://cottonfgd.net/)	2,251,322 -2,252,656
Ghir_A07G008080	*GhTT19*	Glutathione S-transferase F12	*G.hirsutum* (https://cottonfgd.net/)	10,653,327 -10,655,660
Gbar_A07G008330	*GbBM*	Transcription factor MYB113	*G.barbadense* (https://cottonfgd.net/)	11,508,142 -11,513,159

Supplementary Table 3 Primers used in this study.

Primer ID	Forward primer 5'-3'	Reverse primer 5'-3'	Purpose
RF1	CTGCACCCTTTCCAATGCTTTT	TCAGTAGTGACATGTGAAGTACTT	Fine mapping
RF2	GGGTTGAAGAGACCCTCCCTC	CGGACAAAATAGCAGTTAATCCTCA	Fine mapping
RF3	AAGATGAAGTCCGGAAGTGAAT	GGTGAGCCCTCTCTTAAATGACA	Fine mapping
RF4	CATAAGTTCGCCTCAGCACGA	AGACAGATCGCCAATTACGTTTG	Fine mapping
RFC1	AATGGGCCTTGAAATCTGTTGA	ACTGTCTGGTTATTCCGTCTGT	Fine mapping
RFC2	ATCCCTCTTACAAATCTCCACC	TGCTGCCATCCTTTTAAGTTTT	Fine mapping
Ghir_A07G008080	ATGGTAGTGAAAGTGTATGGTC	CCGGCTTCGAGATCGACGTC	qRT-pcr
Ghir_A07G008660	ACCATTGGTAACTCCGCAGGAA	ACAGATAAGTCCGCCGCAGAA	qRT-pcr
Ghir_A07G018850	CCTTGTGCGAGTCCACCATCA	GTACCTCTCCCTTGCTTCTCCA	qRT-pcr
Ghir_A07G021890	GACAACAAGGATAAGGTAGC	AGGTCTCTGCGTCCAATA	qRT-pcr
Ghir_D07G008160	CGAGCAATGGTGGATCAATGGC	AGCAAGATAGGCGGTGGTGGA	qRT-pcr
Ghir_A10G021520	GGTGCCCGTGATATTCTTGGAGAC	TCAGAATCAGCCACCCATTTGTCA	qRT-pcr
Ghir_A02G003240	CGATGTCATCCACCTCCCACCA	ACGAGAGCAGTTGGCAGAGTCT	qRT-pcr
Ghir_A02G002860	GAAGAACAACGAGCGGCAGTGA	CCAAGCCAGTGAGCAACAAAGGA	qRT-pcr
GhHistone	CAGGAAATTGCCTTTCCAGA	TGGATGTCCTTGGGCATAAT	qRT-pcr

Supplementary Table 6 Correspondence between Gene IDs and Gene Names.

Gene ID	Gene Name
Ghir_D12G023690	ANS_D12
Ghir_D12G019530	F3’H_D12
Ghir_D05G019650	DFR_D05
Ghir_DO4G001380	CHI_D04
Ghir_D03G005110	UFGT_D03
Ghir_D02G003050	CHS_D02
Ghir_A12G023690	ANS_A12
Ghir_A12G019280	F3’H_A12
Ghir_A10G018240	C4H_A10
Ghir_A10G012390	CHS_A10
Ghir_A09G015780	PAL_A09
Ghir_A07G002090	GhTT2_3A
Ghir_A06G000790	DFR_A06
Ghir_A05G041560	CHI_A05
Ghir_A05G017310	ANR_A05
Ghir_A02G015530	4CL_A02
Ghir_D12G027910	LAR_D12
Ghir_A12G018100	LAR_A12
Ghir_A02G015500	UFGT_A02
